# FLASH Irradiation Modulates Immune Responses and Accelerates Lung Recovery: A Single‐Cell Perspective

**DOI:** 10.1002/advs.202501797

**Published:** 2025-06-19

**Authors:** Hao Lu, Menghua Li, Cheng Quan, Caihui Li, Dawei Li, Zhihui Li, Jing Xu, Lihui Zhang, Qixiang Liu, Guofu Dong, Changzhen Wang

**Affiliations:** ^1^ Beijing Institute of Radiation Medicine Beijing 100850 P. R. China; ^2^ Anhui Medical University Hefei 230032 P. R. China

**Keywords:** conventional dose rate irradiation, FLASH irradiation, immune regulation, radiation‐induced lung injury, single‐cell RNA sequencing

## Abstract

Radiation therapy (RT) is essential for treating thoracic malignancies but often causes significant lung damage. FLASH‐RT, an ultra‐high dose rate irradiation technique, shows potential in reducing radiation‐induced lung injury (RILI) while maintaining tumor control. However, the underlying immune mechanisms remain poorly understood. This study investigates the immune and cellular responses to FLASH‐RT versus conventional dose rate (CONV) RT during the early phase of RILI. Using single‐cell RNA sequencing (scRNA‐seq), a dynamic landscape of the lung microenvironment is pictured during RILI within one‐week post‐irradiation. The analysis reveals that FLASH‐RT induces a more immediate but transient cellular response, while CONV‐RT causes sustained inflammation. FLASH irradiation significantly reduces neutrophil infiltration compared to CONV irradiation, particularly within the pro‐inflammatory Ccrl2^+^ subset. FLASH irradiation also triggers stronger activation of CD4^+^ CD40L^+^ Th cells, which are critical for regulating immune responses and balancing inflammation. Moreover, FLASH irradiation attenuates pro‐inflammatory activation and intercellular signaling of Mefv⁺ monocytes, thereby restraining excessive macrophage‐driven inflammation. Additionally, FLASH irradiation enhances TGF‐β signaling and epithelial‐mesenchymal transition (EMT) in alveolar type 1 (AT1) cells, promoting tissue repair. These findings highlight FLASH‐RT's superior immune modulation and reparative potential, providing valuable insights into its clinical application for minimizing radiation damage and enhancing lung recovery.

## Graphical abstract

Single‐cell RNA sequencing reveals distinct immune responses to FLASH versus conventional dose rate irradiation in early radiation‐induced lung injury. FLASH irradiation reduces Ccrl2⁺ neutrophil infiltration, activates CD4⁺ CD40L⁺ Th cells, restrains pro‐inflammatory Mefv⁺ monocytes, and enhances epithelial repair via TGF‐β signaling, underscoring its therapeutic potential in mitigating radiation‐induced lung damage.

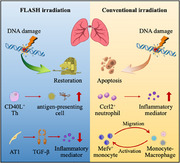

## 1 Introduction

Radiation therapy (RT) is a widely utilized approach for treating thoracic malignancies, yet it is often accompanied by clinical complications such as impaired wound healing in irradiated tissues and damage to surrounding normal lung tissues.^[^ ^1^, ^2^ ^]^ These adverse effects have spurred an urgent demand for advancements in RT technologies and strategies that can effectively protect normal tissues.^[^ ^3^, ^4^ ^]^ Over the past decade, ultra‐high dose rate (UHDR) pulsed irradiation (mean dose rate ≥40 Gy s^−1^) has demonstrated promising “FLASH” effects, significantly reducing damage to normal tissues while preserving tumor control efficacy compared to conventional dose rates (CONV, 0.001 to 0.4 Gy s^−1^).^[^ ^5^, ^6^, ^7^, ^8^, ^9^ ^]^ Recent studies further report enhanced control of radioresistant tumors and greater suppression of tumor growth under FLASH irradiation.^[^ ^10^, ^11^ ^]^ The protective FLASH effects have been observed in multiple tissues, including the brain, lungs, intestine, and skin.^[^ ^12^, ^13^, ^14^, ^15^ ^]^ However, challenges remain in translating this technique into clinical practice, particularly due to an incomplete understanding of the biological mechanisms, especially immune modulation, that mitigate radiation‐induced damage.^[^ ^16^, ^17^ ^]^ Besides, most research has focused on immune responses within tumors, with little attention paid to immune dynamics in irradiated normal tissues.

Given the significant immune involvement in radiation‐induced lung injury (RILI), understanding how immune mechanisms are leveraged in FLASH‐RT is crucial for mitigating these adverse effects. Previous studies have shown that FLASH‐RT reduces inflammatory immune cell infiltration and pro‐inflammatory cytokine secretion, alleviating toxicity in multiple tissues.^[^ ^15^, ^18^, ^19^, ^20^, ^21^ ^]^ For example, FLASH‐RT decreases macrophage and neutrophil accumulation and prevents lymphocyte infiltration, thereby reducing both acute pneumonitis and late‐stage lung fibrosis.^[^ ^15^, ^22^ ^]^ FLASH‐RT also protects against radiation‐induced apoptosis by suppressing TGF‐β/SMAD activation in blood vessels and bronchi, reducing pro‐inflammatory gene expression in normal lung tissues.^[^ ^15^ ^]^ However, the impact of FLASH‐RT on the cellular microenvironment and intercellular interactions, as well as their dynamic changes, particularly during the early phases of injury in irradiated normal lung tissues, remains poorly understood.

In this study, we address these challenges by performing single‐cell RNA sequencing (scRNA‐seq) on lung tissue samples from mice exposed to either CONV or FLASH‐RT. Our goal is to investigate the early immunological distinctions in RILI between the two modalities and to identify potential immune mechanisms underlying the protective FLASH effect. By profiling over 135 000 single cells from the pulmonary microenvironment, we identified key immune and stromal cell dynamics distinguishing RILI processes in CONV and FLASH‐RT. These findings offer valuable insights into the biological mechanisms of FLASH‐RT and its potential clinical applications in thoracic malignancies.

## 2 Results

### 2.1 A Dynamic Single‐Cell Atlas of Injured Lung Post‐Conventional (CONV) and FLASH Irradiation

**Figure 1:**
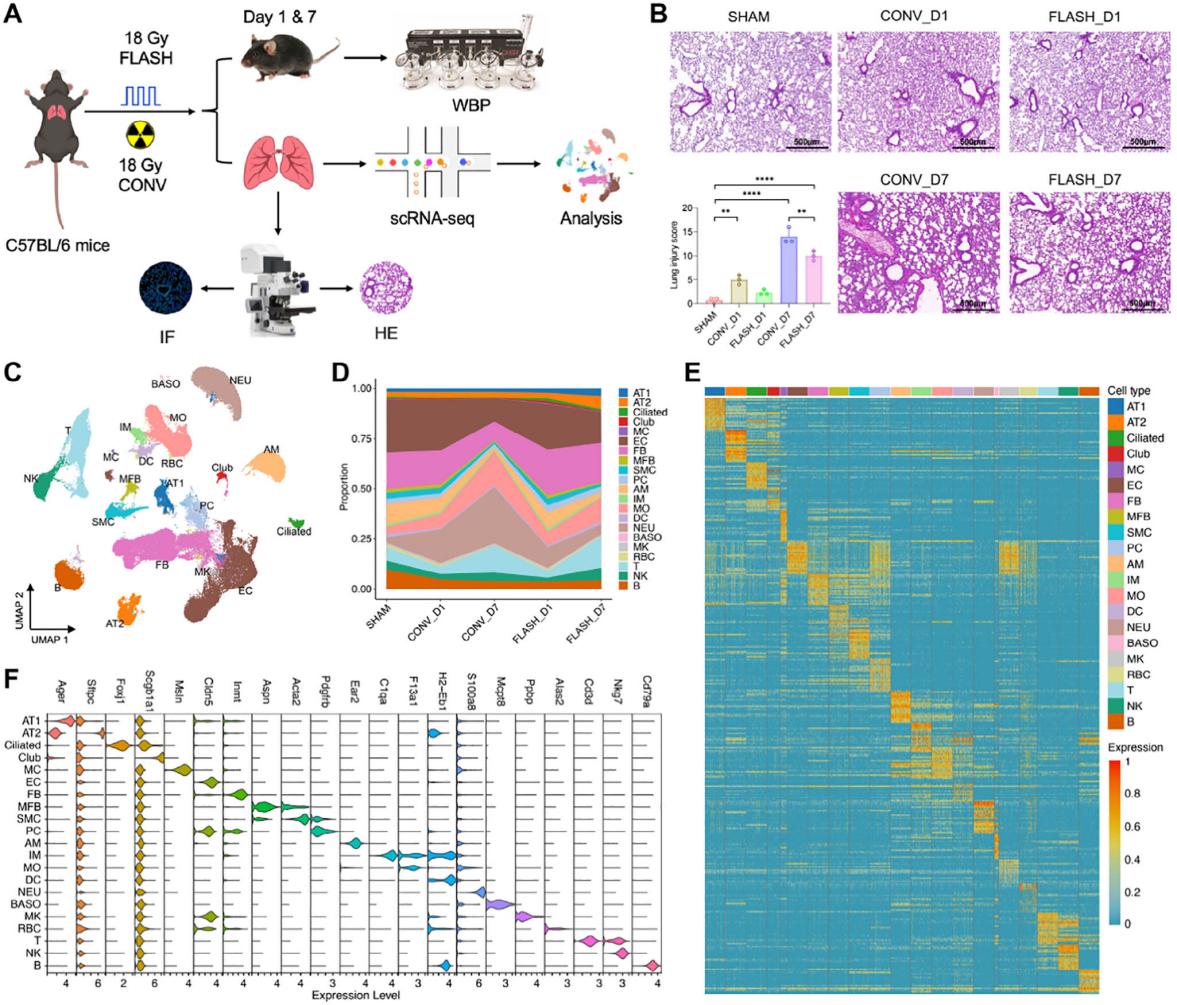
Construction of a cellular atlas of mouse RILI following CONV and FLASH irradiation. A) Flow chart of the experimental design and analysis. B) Representative images and Szapiel scoring of H&E staining results of lung tissue sections at 1 day and 7 days post‐irradiation (*n* = 3, scale bar = 500 µm). ^**^ *p* <0.01, ^****^ *p* <0.0001. C) UMAP projection of the 135 633 cells profiled, colored by annotated cell types. D) Area chart showing the dynamic changes of the proportions of cell types in healthy lung samples (SHAM) and exposed lung samples at different times after CONV and FLASH irradiation. E) Heatmap showing the expression of top 20 differentially expressed genes (DEGs) in each cell type. For visualization, 1000 cells were randomly selected from cell types with a total count exceeding 1000. F) Vlnplot showing the expression of representative marker genes in each cell type. AT1, alveolar type 1; AT2, alveolar type 2; MC, mesothelial cell; EC, endothelial cell; FB, fibroblast; MFB, myofibroblast; SMC, smooth muscle cell; PC, pericyte; AM, alveolar macrophage; IM, interstitial macrophage; MO, monocyte; DC, dendritic cell; NEU, neutrophil; BASO, basophil; MK, megakaryocyte; RBC, red blood cell; NK, natural killer cell.

To construct the RILI model, C57BL/6J mice were exposed to 17.8 Gy whole‐thorax irradiation delivered in the FLASH or CONV mode (**Figure** **1**A). The dose was selected based on prior studies, including the seminal work by Favaudon et al.,^[^ ^15^ ^]^ which demonstrated that 18 Gy of thoracic FLASH irradiation in mice induces moderate fibrotic changes without lethality 24 weeks post‐irradiation. This dose also falls within the recognized tolerance range of murine lung tissue (18–20 Gy),^[^ ^23^ ^]^ allowing assessment of both acute and subacute injury phases. The actual dose delivered was 17.8 Gy due to technical constraints of the irradiation system. Additionally, increasing evidence suggests a nonlinear relationship between dose rate and biological effects, with FLASH irradiation (>40 Gy s^−1^) providing protection to normal tissues beyond a specific threshold dose rate.^[^ ^15^, ^24^ ^]^ This nonlinear response is central to the differential effects observed between FLASH and CONV irradiation. The lung pathology and respiratory function of irradiated mice were assessed to explore the protective effects provided by FLASH irradiation. Whole‐body plethysmography showed that the negative impact of CONV irradiation on pulmonary function was significantly spared by FLASH irradiation. At 1 day post‐irradiation (dpi), both the CONV and FLASH irradiated mice exhibited a significant decrease in the frequency (F), tidal volume (TV), and minute volume (MV), along with a significant increase in expiratory time (Te) (Figure S1A,C–E, Supporting Information). However, compared to the control group, only CONV‐irradiated mice showed a significant reduction in mid‐expiratory flow (EF50), peak inspiratory flow (PIF), and peak expiratory flow (PEF), while these parameters remained unchanged in FLASH‐irradiated mice (Figure S1B,F,H, Supporting Information). CONV irradiation, rather than FLASH irradiation, had a more pronounced impact on pulmonary function in mice at 7 dpi. CONV‐irradiated mice exhibited significant reductions in five parameters (TV, MV, EF50, PIF, and PEF), while inspiratory time (Ti) and Te showed significant increases compared to the control group (Figure S1A,B,E,F,H,G, Supporting Information). In contrast, the FLASH‐irradiated mice did not show significant changes in these parameters. Although both groups exhibited a significant decrease in F and an increase in Te, the variation amplitude of F and Te in FLASH‐irradiated mice was significantly reduced compared to the CONV‐irradiated mice (Figure S1C,D, Supporting Information). Histological analysis with hematoxylin and eosin (H&E) staining confirmed the above results. Although all irradiated lung sections showed areas of cell infiltration, lungs from CONV‐irradiated mice exhibited more severe pathology, with thickened alveolar septa and cellular accumulation as compared to FLASH‐irradiated lungs. The total lung injury scores were significantly higher in CONV‐irradiated mice than in FLASH‐irradiated mice at 7 dpi (Figure 1B), indicating that CONV irradiation caused more severe lungpathology.

To evaluate whether these early differences translate into long‐term effects, we examined lung tissues at 1, 3, and 6 months post‐irradiation. H&E staining showed progressive lung injury in both groups, but the FLASH group had less inflammatory infiltration and septal thickening at all time points (Figure S2A, Supporting Information). At 6 months, the CONV group showed severe alveolar collapse and loss of normal architecture, while the FLASH group retained relatively preserved alveolar structures. Masson staining revealed significantly less collagen accumulation in the FLASH group, especially at 3 and 6 months (Figure S2B, Supporting Information), suggesting attenuated fibrosis. These results indicate that FLASH irradiation not only mitigates acute injury but also confers long‐term structural protection.

Subsequently, single‐cell RNA sequencing (scRNA‐seq) was performed to investigate the mechanisms underlying the differential responses to CONV and FLASH irradiation. After quality control, we obtained transcriptomic data from 135 633 high‐quality cells across 25 611 genes from 15 samples, with each cell expressing an average of 3067 genes and containing 10 245 unique molecular identifiers (UMIs) (Figure S3A–D, Supporting Information). Unsupervised clustering and cell annotation revealed 20 distinct cell types or states based on the expression of canonical markers (Figure 1C–F; Figure S3C, Supporting Information), including six myeloid cell types, three lymphoid cell types, three mesenchymal cell types, five epithelial cell types, and endothelial cells, with no discernible batch effects on data architecture. Comparison between different groups revealed that cellular compositions changed markedly during the CONV and FLASH‐induced injury process (Figure 1D). Myeloid cells, primarily neutrophils and monocytes, exhibited a dramatic increase at 7 dpi post‐CONV irradiation, whereas FLASH irradiation did not elicit a similar response. Lymphoid cells demonstrated a moderate decrease at 1 dpi and an increase at 7 dpi in both CONV and FLASH groups. Endothelial and mesenchymal cells showed a relative decrease at 7 dpi in both irradiation groups. Overall, these results provide a comprehensive single‐cell atlas of the healthy and injured lungs following CONV and FLASH irradiation and suggest a significant difference between the immune microenvironment of the two irradiation types during the early phase of RILI. To facilitate interactive exploration of the cellular microenvironment of RILI induced by the two irradiation modalities, we created a web interface at http://Flash‐RILI.omic.tech.

### 2.2 Impact of CONV and FLASH Irradiation on Lung Cell Composition and Radiosensitivity

**Figure 2:**
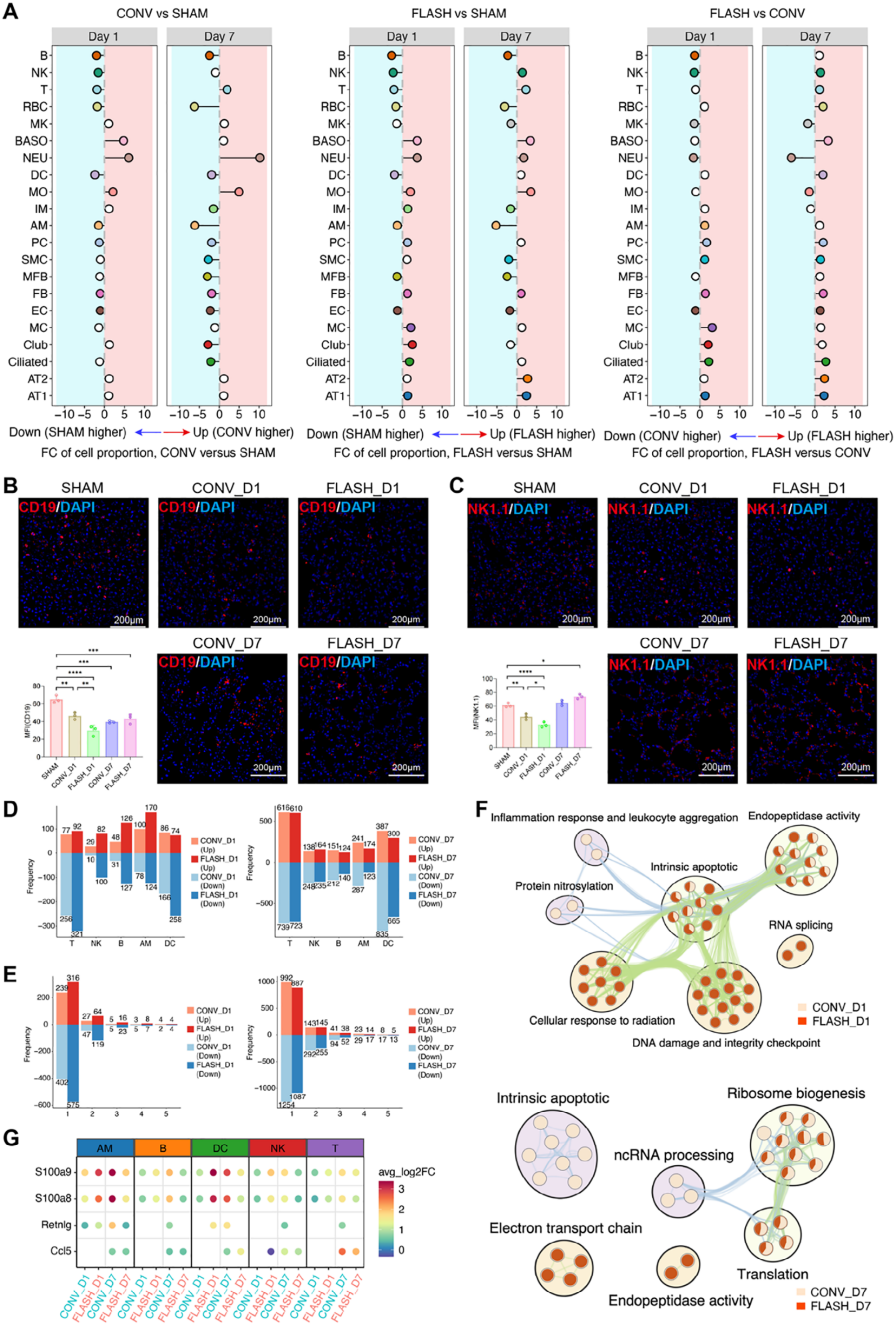
Characteristics of in vivo radiosensitivity of different cell types to CONV and FLASH irradiation. A) Lollipop plot showing the proportional differences of each cell type across three comparisons. B,C) Multiplex immunostaining in lung sections showing the expression of CD19 (red) (B) and NK1.1 (red) (C) following CONV and FLASH irradiation. The embedded bar plot shows the quantification of expression of CD19 and NK1.1 in lung tissues of each group after irradiation (*n* = 3, scale bar = 200 µm). ^*^ *p* <0.05, ^**^ *p* <0.01, ^***^ *p* <0.001, ^****^ *p* <0.0001. D) Bar plot denoting the number of DEGs in each group on Day 1 (left) and Day 7 (right) compared to SHAM. E) Bar plot denoting the categorization of DEGs according to the number of shared tissues in each group on Day 1 (left) and Day 7 (right) compared to SHAM. F) The top significantly enriched GO biological process terms (adjusted *p* <0.001) of the common DEGs on Day 1 (top) and Day 7 (bottom) compared to SHAM. G) Dot plot showing the log2FC values of the selected markers in common DEGs.

The dynamic changes in the cellular composition of the lung microenvironment before and after CONV and FLASH irradiation offer valuable insights into the radiosensitivity of various cell subtypes in vivo. We quantified the radiosensitivity of each cluster by comparing the proportions of cell types across different groups. Consistent with our previous studies,^[^ ^25^ ^]^ immune cells, particularly lymphoid cells, were highly sensitive to CONV irradiation, showing significantly reduced proportions at 1 dpi (**Figure** **2**A; Figure S4A,B, Supporting Information). Notably, FLASH irradiation induced an even greater decrease in lymphoid cells at 1 dpi, including B cells and NK cells. Mesenchymal and endothelial cells exhibited reduced proportions at 7 dpi following CONV irradiation, although this may be attributed to the substantial infiltration of neutrophils post‐irradiation. While FLASH irradiation similarly caused a reduction in mesenchymal and endothelial cells, the effect was less pronounced compared to CONV irradiation. Immunostaining confirmed significant reductions in B and NK cell numbers at 1 dpi for both irradiation types, with FLASH causing a more substantial decrease. By 7 dpi, no significant differences were observed between the two irradiation types (Figure 2B,C).

We then aimed to identify the specific signatures and pathways activated in irradiation‐sensitive cell types in response to radiation‐induced apoptosis. Comparative analysis between the irradiated and SHAM groups revealed numerous differentially expressed genes (DEGs) across various cell types. Of note, at 1 dpi, FLASH irradiation induced a greater number of DEGs compared to CONV irradiation (Figure 2D), suggesting that FLASH triggers more pronounced early cellular responses. However, by 7 dpi, CONV irradiation resulted in more DEGs, indicating a stronger and more prolonged impact of CONV in the later phase of the response (Figure 2D). Correspondingly, we found more common DEGs shared by at least three cell types in the FLASH group at 1 dpi and in the CONV group at 7 dpi, respectively (Figure 2E; Table S1, Supporting Information). Gene Ontology (GO) term annotation of common DEGs revealed that at 1 dpi, the common DEGs in the FLASH group were enriched in pathways related to cellular response to irradiation, DNA damage, and intrinsic apoptosis, while those in the CONV group were associated with inflammatory response, leukocyte aggregation, and protein nitrosylation (Figure 2F). By 7 dpi, the FLASH group was linked to the electron transport chain and endopeptidase activity, while the CONV group was predominantly involved in intrinsic apoptosis and translation pathways (Figure 2F). The most common genes include the inflammation mediators, such as *S100a8*, *S100a9*, and *Retnlg*, and the chemokine *Ccl5* (Figure 2G). These results indicate that FLASH irradiation may elicit a more immediate cellular response to radiation‐induced damage, while CONV irradiation appears to have a more significant and sustained effect in the later stages.

### 2.3 Accumulation of Ccrl2^+^ Neutrophils Promotes the Inflammatory Response Post‐CONV Irradiation

**Figure 3:**
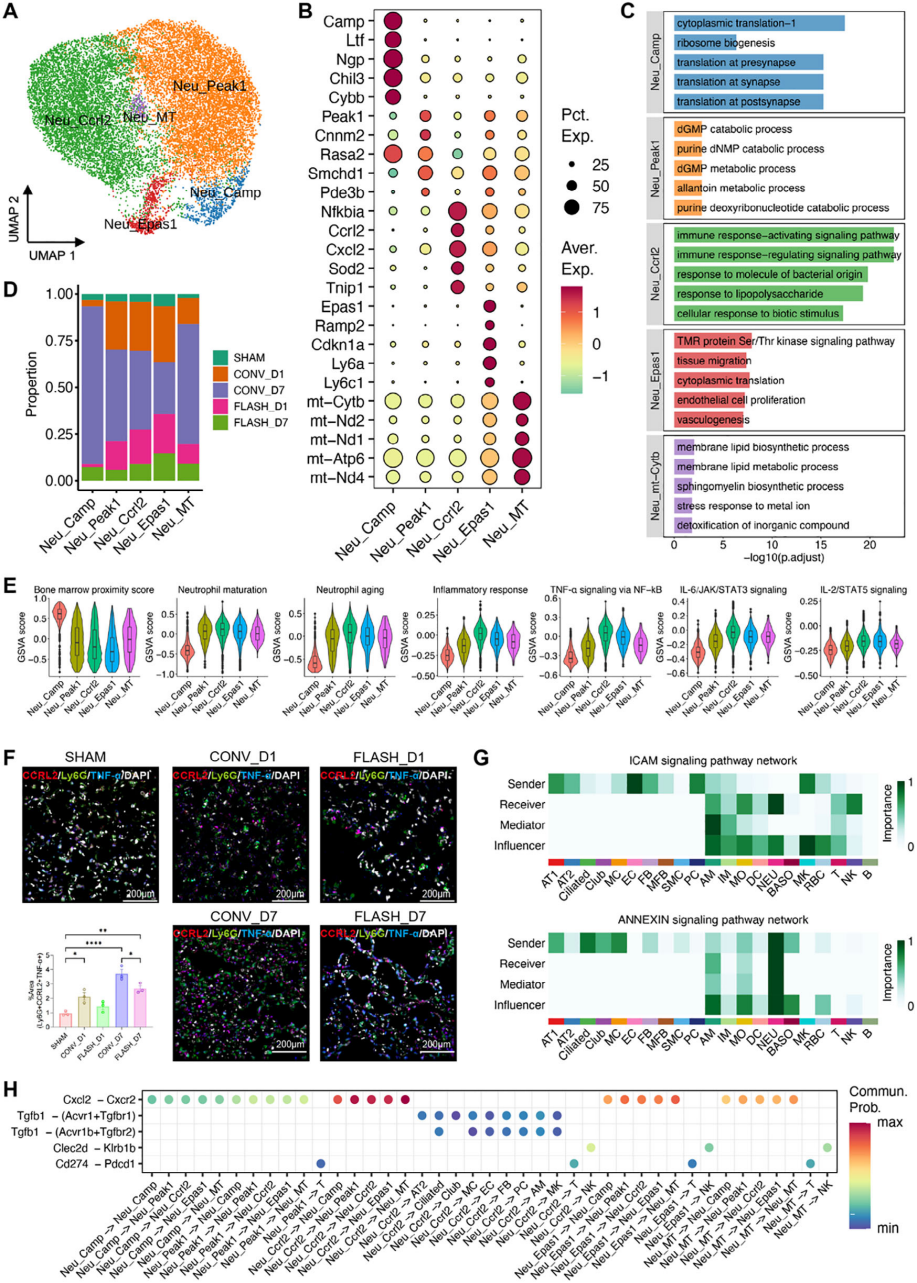
Neutrophils exhibited overwhelming infiltration following CONV irradiation. A) UMAP projection of 17 191 neutrophils identified, colored by cluster identities. B) Expression of selected marker genes in each neutrophil subtype. C) The top five significantly enriched GO biological process terms of the marker genes in each neutrophil subtype. D) Bar plots showing the fraction of cells originating from different groups in each cluster. E) Violin plots showing GSVA scores of selected pathways in the neutrophil clusters. For each embedded boxplot, the box represents the interquartile range, the horizontal line in the box indicates the median, and the whiskers represent 1.5 times the interquartile range. F) Multiple immunostaining showing co‐expression of Ly‐6G (green), Ccrl2 (red) and TNF‐α (blue) in lung tissue sections after CONV and FLASH irradiation. Embedded bar graphs show the quantification of Ly‐6G, Ccrl2 and TNF‐α co‐localization in lung tissues of irradiated groups (*n* = 3, scale bar = 200 µm). ^*^ *p* <0.05, ^**^ *p* <0.01, ^****^ *p* <0.0001. G) Heatmap depicting the relative importance of each cell type as sender, receiver, mediator and influencer in ICAM and ANNEXIN signaling pathways. H) Dot plot showing the specific ligand‐receptor pairs associated with the Ccrl2^+^ neutrophil subset.

Having observed significant differences in neutrophil infiltration between CONV and FLASH irradiation, we analyzed the distinct transcriptomic profiles of neutrophils under these conditions. A total of 17 191 neutrophils were identified in our dataset, categorized into five subpopulations (**Figure** **3**A). We delineated marker genes for each cluster and associated them with specific functions (Figure 3B,C). This analysis revealed five distinct clusters: antimicrobial neutrophils (Neu_Camp), characterized by high expression of granule genes (*Camp*, *Ltf*, and *Ngp*) and activation in translation and biogenesis processes; activated or mature neutrophils (Neu_Peak1), showing elevated expression of signaling transduction and metabolic regulation mediators (*Peak1*, *Cnnm2*, and *Pde3b*), consistent with their enrichment in dGMP and purine catabolic pathways; inflammatory neutrophils (Neu_Ccrl2), exhibiting high expression of inflammatory and redox molecules (*Nfkbia*, *Ccrl2*, and *Sod2*), in line with activation in immune response pathways; and two clusters indicating stress‐induced states, one marked by a hypoxia‐induced signature (*Epas1*, *Ramp2*, and *Cdkn1a*) and enriched in vasculogenesis processes, and another marked by mitochondrial activity signatures (*mt‐Cytb*, *mt‐Nd2*, and *mt‐Nd1*) and showing activation in membrane lipid biosynthetic and metabolic processes.

Exploration of the differences in group distribution revealed that all neutrophil subtypes exhibited remarkably increased fractions after both CONV and FLASH irradiation, with more dramatic increases in CONV than FLASH group, especially at 7 dpi (Figure 3D). To further elucidate the phenotypic differences among neutrophil subsets and the sources of inflammation, we calculated pathway scores related to neutrophil functions and inflammatory responses for each cell based on the expression of corresponding signatures. This analysis revealed that Neu_Camp had higher bone marrow proximity scores, while the other four clusters showed higher maturation and aging scores, reflecting their distinct maturation states (Figure 3E). Notably, Neu_Ccrl2 displayed the highest scores in inflammatory pathways, including inflammatory responses, IL‐2/STAT5 signaling, and IL‐6/JAK/STAT3 signaling (Figure 3E), suggesting its pivotal role in orchestrating inflammation during the early phase of RILI. Immunofluorescence staining confirmed neutrophil infiltration in lung tissues in both irradiated groups. The numbers of total neutrophils and Ccrl2^+^ TNF‐α^+^ neutrophils increased in both groups, with the CONV group showing approximately twice the increase compared to the FLASH group at 7 dpi (Figure 3F).

To investigate the potential role of neutrophil populations in the microenvironment of LIRI, we examined cell‐cell communication (CCC) processes across different groups. The results indicated that the ICAM and ITGAL‐ITGB2 signaling pathways, which mediate neutrophil adhesion and migration,^[^ ^26^ ^]^ were significantly more active at 7 dpi in the CONV group compared to those in the SHAM and FLASH groups (Figure 3G; Figure S5A,B, Supporting Information). Furthermore, ANNEXIN^[^ ^27^ ^]^ and LAIR1,^[^ ^28^ ^]^ which exert anti‐inflammatory functions in neutrophils, also exhibited stronger activation in the CONV group (Figure 3G; Figure S5B,C, Supporting Information), suggesting the involvement of a negative feedback regulatory mechanism in response to the heightened inflammatory state. Analysis of neutrophil subsets revealed elevated activation of chemokines and TGF‐β signaling in Ccrl2^+^ neutrophils (Figure 3H). We found that Ccrl2^+^ neutrophils significantly enhanced the recruitment of other subsets through the Cxcl2‐Cxcr2 interaction. Additionally, they would promote the activation of epithelial and mesenchymal cells via TGF‐β signaling, which may contribute to pulmonary fibrosis following irradiation exposure.^[^ ^29^ ^]^

### 2.4 Transcriptional Evolution of Neutrophils During Radiation‐Induced Injury

**Figure 4:**
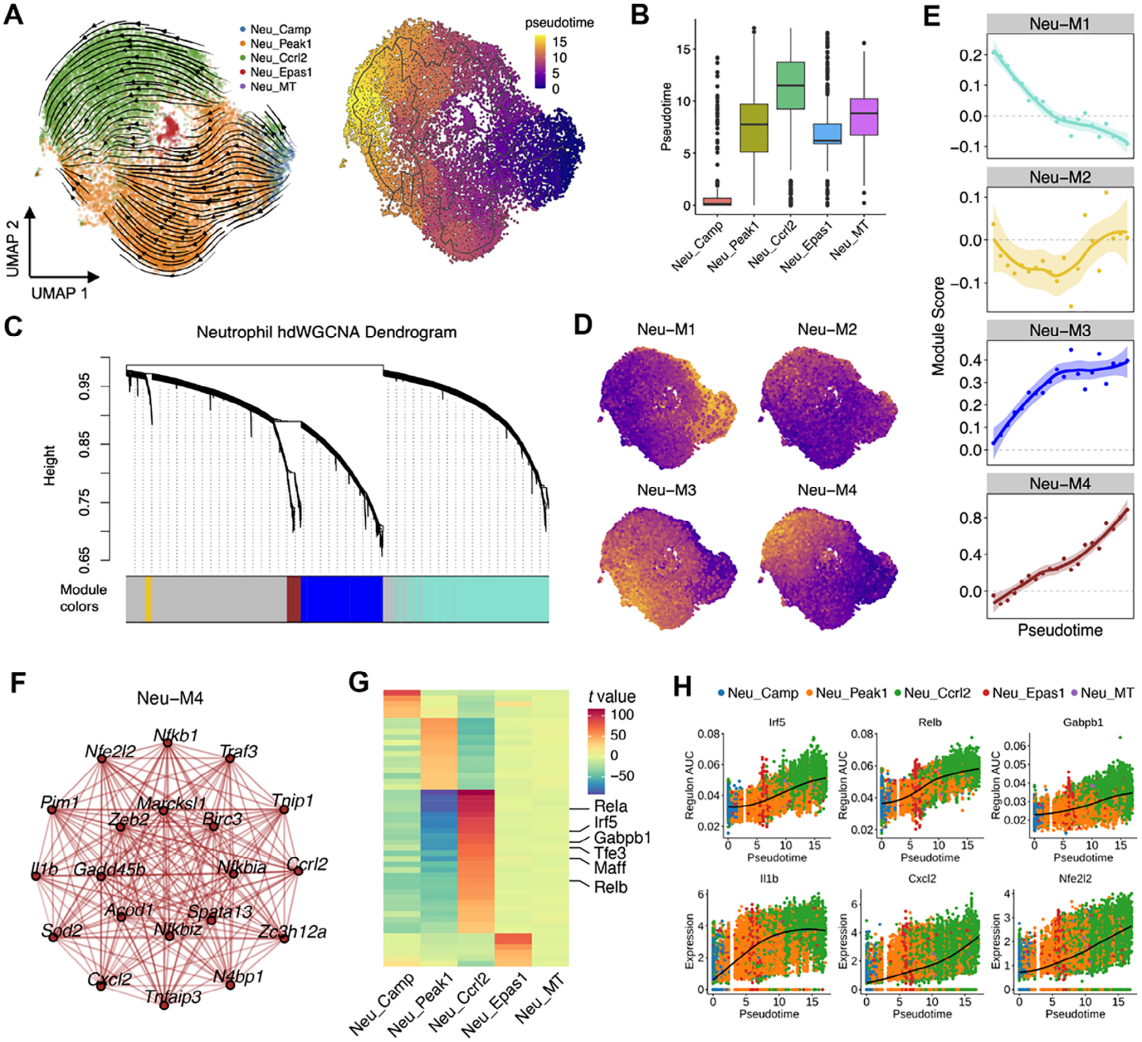
Transcriptional evolution of diverse neutrophil populations during RILI. A) Neutrophil differentiation routes (left) and pseudotime scores (right) revealed by trajectory analysis. The trajectory flows were inferred from scVelo and the UMAP dimension reduction and pseudotime estimation were performed using monocle V3. B) Boxplots showing the distribution of pseudotime scores among five neutrophil subtypes. C) Hierarchical clustering dendrograms of identified co‐expressed genes and the associated modules in neutrophils. D) Distribution of module scores across neutrophil subtypes. Module scores were estimated by AddModuleScore in Seurat. E) Dot plots depicting the correlation of module scores with pseudotime scores in neutrophils. F) Co‐expression network of the top 20 hub genes in module 4. G) Differences in the activity of the top 50 regulon among neutrophil subsets. Shown are *t* values calculated in a linear model comparing the regulon scores estimated by SCENIC between cells from one cluster and those from all other clusters. H) Dynamic activity of the selected TF regulons and expression of the regulated genes along the pseudotime.

To investigate neutrophil phenotypic conversion during RILI, single‐cell trajectory analysis was performed using Monocle^[^ ^30^ ^]^ and RNA velocity.^[^ ^31^ ^]^ This analysis indicated a clear velocity flow from Neu_Camp to Neu_Peak1 and Neu_Ccrl2, signifying a transition from myeloid‐derived neutrophils to mature neutrophils (**Figure** **4**A). Neu_Ccrl2 exhibited the highest pseudotime scores, suggesting a terminal differentiation state (Figure 4B). We subsequently employed weighted gene co‐expression network analysis (WGCNA)^[^ ^32^ ^]^ to examine gene expression changes underlying the differentiation trajectory, identifying four gene modules with distinct dynamic patterns (Figure 4C; Figure S6A, Supporting Information). Notably, module 4 showed a continuous increase in module score along the trajectory and was specifically activated in the Ccrl2^+^ subset (Figure 4D–F). Functional annotation of module genes revealed significant enrichment of cytokine signaling, oxidative damage response, and intrinsic apoptotic signaling in module 4 (Figure S6B, Supporting Information), indicating a state of highly activated inflammation and oxidative stress. To explore key regulons along the transition flow, we applied Single‐Cell Regulatory Network Inference And Clustering (SCENIC),^[^ ^33^ ^]^ uncovering numerous specific regulons in each neutrophil population (Table S2, Supporting Information). We found that transcription factors (TFs) involved in immune response regulation, such as Irf5, Relb, and Rela, were significantly activated in Ccrl2^+^ neutrophils (Figure 4G). Irf5 and Relb have been shown to orchestrate neutrophil activation and effector functions by regulating the expression of inflammatory mediators, including *Il1b*, *Tnfa*, and *Cxcl2*,^[^ ^34^ ^]^ in response to zymosan treatment. Here our results suggest that Irf5 and Relb may facilitate neutrophil activation by promoting the conversion from granule‐producing neutrophils to pro‐inflammatory ones following radiation‐induced injury, particularly in response to CONV irradiation. Additionally, TFs associated with cell metabolism and mitochondrial function, such as Gabpb1, Tfe3, and Maff, also exhibited high activation in this subset. Of note, The *Gabpb1* gene encodes the beta1 subunit of Nfe2l2 (a.k.a. Nrf2) protein and activates certain mitochondrial enzymes responsible for antioxidant action and detoxification.^[^ ^35^ ^]^ Our findings suggest a novel role for *Gabpb1* in Ccrl2^+^ neutrophils, where its activation may significantly enhance the cell survival capacity under oxidative stress during radiation‐induced injury. Moreover, all identified regulons demonstrated significantly increased expression along predicted pseudotime trajectories (Figure 4H). These results elucidate the potential transitional process among diverse neutrophil populations following lung injury and identify specific TF sets driving these differentiation paths.

### 2.5 FLASH Induced Higher Immune Response but Lower Inflammation in T Cells

**Figure 5:**
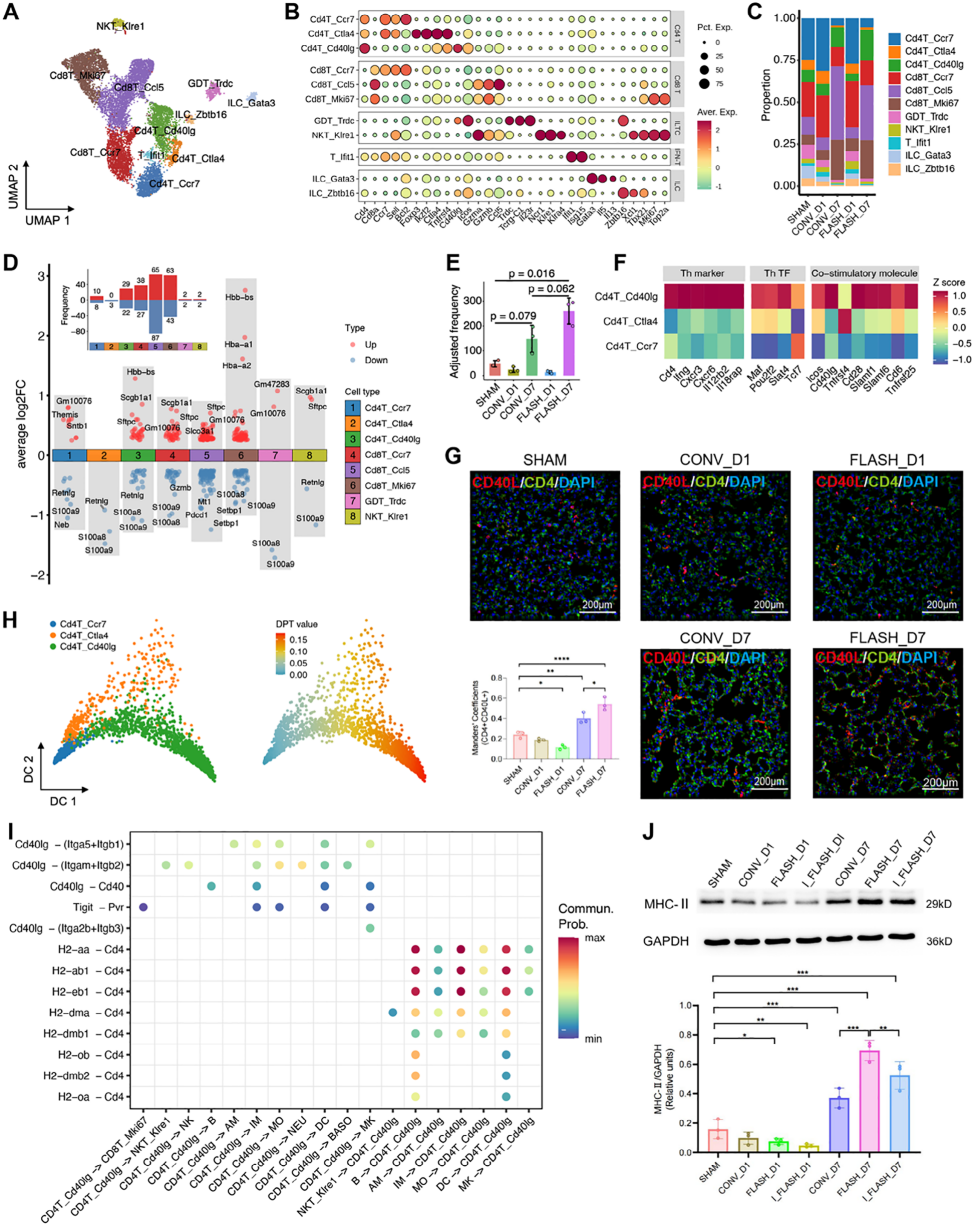
Dynamics of T cell subtypes during lung injury induced by CONV and FLASH irradiation. A) UMAP projection of 11 536 T cells identified, colored by cluster identities. B) Expression of selected marker genes in each T cell subtype. C) Bar plots showing the fraction of cells from different clusters in each group. D) Dot plot showing the DEGs in each cluster between FLASH and CONV irradiation at 7 dpi. The embedded bar plot shows the number of DEGs. E) The adjusted frequency of CD4T_Cd40lg cells in each group. Cell numbers were scaled to a total of 10 000 per sample. F) Heatmap of the scaled average expression of the selected Th function‐related markers in three CD4^+^ T clusters. G) Multiplex immunostaining in lung sections showing the expression of CD4 (green) and CD40L (red) post‐CONV and FLASH irradiation. The embedded bar plot shows the quantification of CD4 and CD40L colocalization in lung tissues of each group after irradiation (*n* = 3, scale bar = 200 µm). ^*^ *p* <0.05, ^**^ *p* <0.01, ****p <0.0001. H) Projection of CD4^+^ T cells on the first 2 diffusion components (DCs), colored by cluster identities (left) and the diffusion pseudotime values (right). I) Dot plot showing the specific ligand‐receptor pairs associated with the CD4^+^ CD40L^+^ T subset. J) WB results showing the levels of MHC‐II in the lung tissues of mice in each group after irradiation, with embedded bar graphs indicating the relative expression of MHC‐II (*n* = 3). ^*^ *p* <0.05, ^**^ *p* <0.01, ^***^ *p* <0.001.

T cells play a crucial role in the regulation of inflammation following lung injury; however, their phenotypic and dynamic differences after CONV and FLASH irradiation have not been thoroughly characterized. In this study, we observed an increase in T cell populations in both irradiation groups and aimed to explore their functions in greater detail. Our dataset identified 11 536 T cells, which were categorized into 11 clusters: three for CD4^+^ T cells, three for CD8^+^ T cells, two for innate‐like T cells (ILTC), one for interferon (IFN)‐stimulated T cells, and two for innate lymphoid cells (ILC) (**Figure** **5**A,B). Analysis of cellular composition revealed that the CD8T_Ccl5, CD8T_Mki67, and CD4T_Cd40lg clusters exhibited a significant increase at 7 dpi in both CONV and FLASH irradiation groups (Figure 5C).

A comparison of transcriptome profiles between CONV and FLASH irradiation identified dozens of DEGs within each cluster at 7 dpi (Figure 5D). Notably, several common DEGs were identified across different T cell clusters (Table S3, Supporting Information). This includes the epithelial markers *Scgb1a1* and *Sftpc*, as well as the hemoglobin genes *Hbb‐b1*, *Hba‐a1*, and *Hba‐a2*, all upregulated in the FLASH group compared to the CONV group. *Scgb1a1* and *Sftpc* play important roles in maintaining the structure and function of the airways and alveoli,^[^ ^36^, ^37^ ^]^ and their upregulation may indicate that T cells promote protective mechanisms in the lungs in response to FLASH irradiation, thereby reducing inflammation and tissue damage. The upregulation of hemoglobin genes suggests an increased demand for oxygen metabolism in T cells, indicating heightened levels of activation and proliferation. Additionally, several genes associated with inflammatory response and leukocyte chemotaxis, including *Retnlg*, *S100a8*, and *S100a9*, showed upregulation in the CONV group, indicating the enhanced function of T cells in promoting the release of inflammatory mediators and recruiting other immune cells. Among the T cell clusters, CD8T_Ccl5 exhibited the most DEGs between FLASH and CONV irradiation (Figure 5D). Functional enrichment analysis revealed that genes upregulated in the FLASH group were primarily enriched in pathways related to the regulation of type II interferon production and hematopoiesis, as well as T cell receptor and activation signaling (Figure S7A, Supporting Information). In contrast, genes upregulated in the CONV group were enriched in pathways associated with responses to external stimuli and T cell apoptosis. These results suggest that FLASH irradiation may be more effective in activating specific immune responses against infections, thereby enhancing the immune system's ability to monitor and clear damaged cells, whereas CONV irradiation may result in widespread tissue damage and subsequent inflammatory responses due to non‐specific stimuli.

CD4^+^ T cells play a crucial role in recognizing exogenous antigens and in regulating and activating CD8^+^ T cells.^[^ ^38^ ^]^ We next explored the dynamics of CD4^+^ T cells following two types of irradiation. Our analysis demonstrated that the CD4T_Cd40lg cluster exhibited a significant increase at 7 dpi after FLASH irradiation, while the cells in the CONV group showed a moderate increase that reached marginal significance (Figure 5E). Examination of their transcriptional profiles revealed that cells in this cluster expressed high levels of helper T (Th) markers and TFs, as well as co‐stimulatory molecules (Figure 5F), indicating that they were engaged in a Th immune response and would actively participate in immune interactions with other immune cells. Immunofluorescence confirmed the presence of CD4 and CD40L co‐expressing T cell subsets. Notably, the number of CD4^+^ CD40L^+^ T cells in lung tissues was significantly higher in both the FLASH and CONV groups compared to the SHAM group at 7 dpi, with FLASH inducing a greater increase than CONV (Figure 5G). Diffusion map dimensionality reduction was applied to explore the relationship between CD4^+^ CD40L^+^ Th cells and other CD4^+^ subsets. This analysis identified a transition trajectory between CD4T_Ccr7 and CD4T_Cd40lg subsets, suggesting that CD4^+^ CD40L^+^ Th cells originate from naïve T cells and are induced by FLASH irradiation (Figure 5H). CCC processes involving different T cell subsets were further investigated to understand their roles in more detail. CD4^+^ CD40L^+^ Th cells were found to be highly involved in immune cell regulation and antigen presentation signaling pathways, including CD40, MHC‐II, TIGIT, and IL‐16 (Figure S7B, Supporting Information). Additionally, these cells stimulate other immune cells through the interaction of CD40L with CD40 and integrins (Figure 5I; Figure S7C, Supporting Information), consistent with their role in enhancing the function of antigen‐presenting cells (APCs).^[^ ^39^, ^40^ ^]^ To validate this, we conducted an in vivo CD40L inhibition experiment. Western blotting (WB) analysis of lung tissues revealed a significant increase in MHC‐II expression in the FLASH irradiation group compared to the CONV group at 7 dpi (Figure 5J). MHC‐II molecules are essential for antigen presentation and the activation of APCs, which play a pivotal role in orchestrating T cell‐mediated immune responses. Notably, after inhibiting the CD40L pathway, MHC‐II expression in the FLASH group was significantly reduced (Figure 5J). These results suggest that CD4+ CD40L+ Th cells, via CD40L‐mediated signaling, are crucial for enhancing the activation of APCs through MHC‐II expression. Additionally, CD4^+^ CD40L^+^ Th cells demonstrated a strong anti‐inflammatory capability by interacting with myeloid cells via the TIGIT/PVR pathway (Figure 5I; Figure S7C, Supporting Information). Overall, these findings suggest that CD4^+^ CD40L^+^ Th cells not only promote immune responses but also help balance pro‐inflammatory and anti‐inflammatory activities, although further studies are still needed to confirm and elucidate the crosstalk processes.

### 2.6 Monocyte and Macrophage Dynamics in Response to CONV and FLASH Irradiation

**Figure 6:**
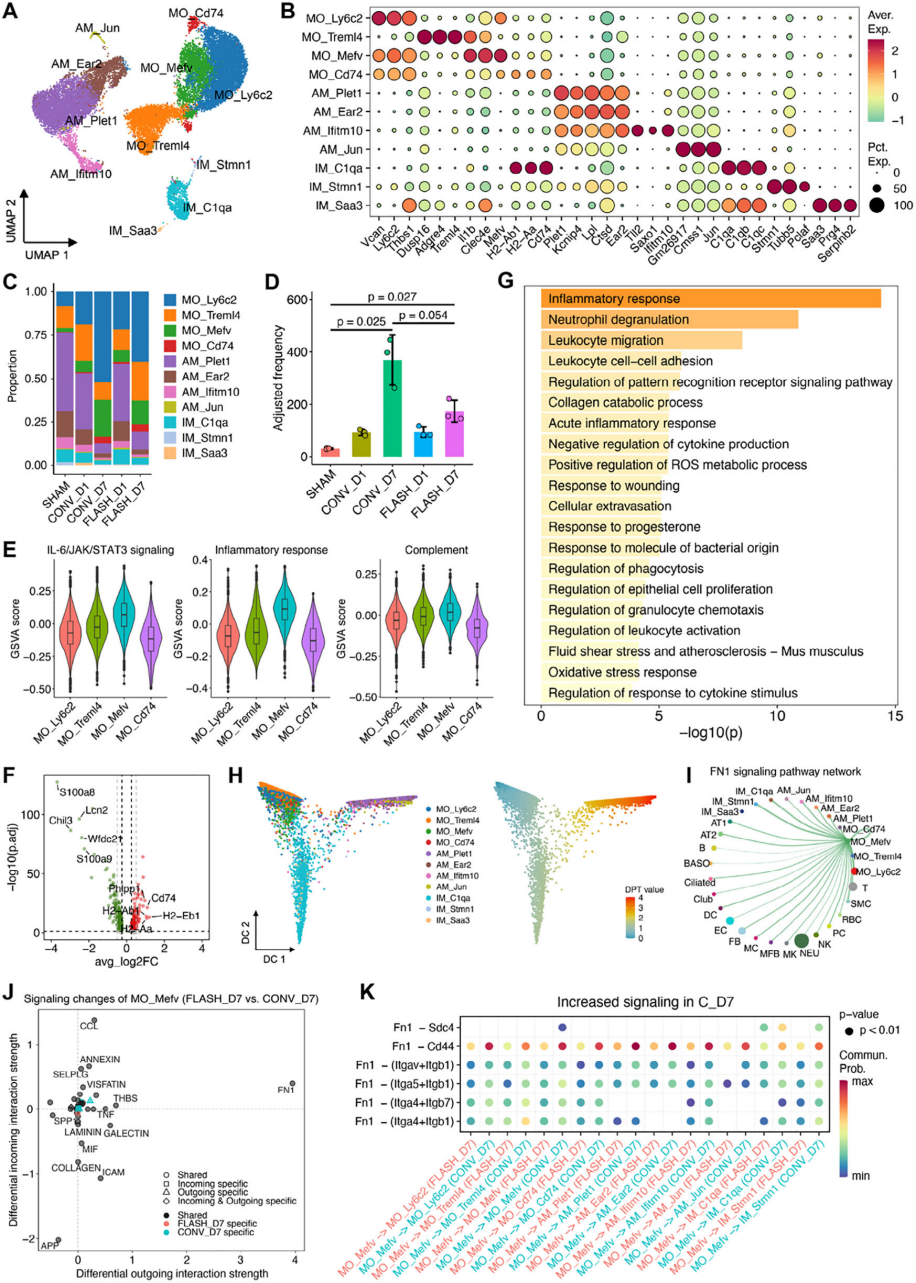
The characteristics of monocyte‐macrophage populations and their cellular interaction in lung injury induced by CONV and FLASH irradiation. A) UMAP projection of 20 642 monocyte–macrophages identified, colored by cluster identities. B) Expression of selected marker genes in each cell subtype. C) Bar plots showing the fraction of cells from different clusters in each group. D) The adjusted frequency of MO_Mefv cells in each group. The number of cells was scaled to a total of 10 000 for each group. E) Violin plots showing GSVA scores of selected pathways in the monocyte clusters. For each embedded boxplot, the box represents the interquartile range, the horizontal line in the box indicates the median, and the whiskers represent 1.5 times the interquartile range. F) Volcano plots showing the DEGs between FLASH and CONV irradiation in MO_Mefv cells at 7 dpi. Vertical lines represent average log2FC thresholds at ±0.25 (black) and ±0.5 (gray), while horizontal lines indicate an adjusted p‐value (p.adj) threshold of 0.05. G) Top 20 significantly enriched signaling pathways of DEGs downregulated in MO_Mefv cells from FLASH compared to CONV group, analyzed using Metascape. H) Diffusion map projection of monocyte‐macrophages on the first 2 DCs, colored by cluster identities (left) and the diffusion pseudotime values (right). I) Dot plot showing the difference of cell‐cell communication (CCC) signaling pathways between CONV and FLASH groups at 7 dpi. J) Circle plot depicting the inferred FN1 signaling pathway network in the CONV group at 7 dpi. K) Dot plot showing the significant ligand‐receptor pairs associated with FN1 signaling pathways.

Among immune cells involved in radiation‐induced lung injury, monocytes and macrophages play a central role by linking early innate immune responses to later tissue remodeling and fibrosis.^[^ ^41^ ^]^ Analysis of 20 642 monocyte‐macrophages across all groups identified 11 clusters: four for monocytes, four for alveolar macrophages (AMs), and three for interstitial macrophages (IMs) (**Figure** **6**A,B). Cell composition analysis revealed a marked increase in monocyte clusters in both CONV and FLASH irradiation groups, accompanied by a relative decrease in AMs and IMs (Figure 6C). Given the pivotal role of macrophage polarization in pulmonary inflammation and tissue repair, M1 and M2 polarization scores were calculated based on established gene signatures.^[^ ^42^ ^]^ AM_Plet1 and IM_C1qa clusters exhibited strong M1 polarization (Figure S8A, Supporting Information). Notably, AM_Plet1 cells from the CONV group displayed higher M1 scores than those from the FLASH group at 7 dpi, despite no significant difference in cell numbers (Figure S8B–D, Supporting Information). Intriguingly, MO_Mefv cells, highly expressing inflammatory and immune modulatory markers such as *Mefv*,^[^ ^43^ ^]^ *Clec4e*,^[^ ^44^ ^]^ and *Il1b*,^[^ ^45^ ^]^ showed substantial infiltration in both CONV and FLASH irradiation groups, with marginally higher infiltration at 7 dpi in the CONV group compared to FLASH (Figure 6D). Hallmark pathway analysis revealed heightened inflammatory response, IL‐6/JAK/STAT3 signaling, and complement activation in MO_Mefv cells (Figure 6E). Furthermore, differential expression analysis showed that MO_Mefv cells from the CONV group were significantly enriched in genes related to inflammatory response and leukocyte adhesion and migration, compared with those from the FLASH group (Figure 6F,G). These results highlight the active role of MO_Mefv in maintaining the pulmonary inflammatory environment.

To investigate lineage relationships, diffusion map analysis was performed, revealing that MO_Mefv resides at the interface between monocytes and IMs, suggesting a transitional state (Figure 6H). CCC analysis showed that MO_Mefv engaged in extensive interactions with other monocyte‐macrophage subsets (Figure S9A, Supporting Information). Compared to FLASH and SHAM, the CONV group exhibited elevated FN1 and CCL signaling (Figure 6I; Figure S9B–D, Supporting Information). FN1 signaling regulates immune cell migration,^[^ ^46^ ^]^ activates stromal cells,^[^ ^47^ ^]^ and promotes fibrotic transformation.^[^ ^48^ ^]^ Notably, MO_Mefv displayed enhanced communication with other myeloid subsets via FN1‐Cd44 and FN1‐integrin interactions (Figure 6J,K), which are known to facilitate adhesion and migration of various myeloid cells.^[^ ^49^, ^50^, ^51^ ^]^ Additionally, MO_Mefv showed increased interactions with other innate immune cells through CCL signaling, particularly via the Ccr1 receptor and its ligands (Figure S9E,F, Supporting Information). Ccr1 signaling modulates monocyte inflammatory responses and promotes differentiation into macrophages.^[^ ^52^ ^]^ Together, these results indicate that MO_Mefv is activated by other innate immune cells and, in turn, contributes to the recruitment and accumulation of additional monocyte‐macrophage subsets, thereby reinforcing the inflammatory microenvironment during the early phase of RILI.

### 2.7 FLASH Irradiation Induces a Robust Repair Response in AT1 Cells

**Figure 7:**
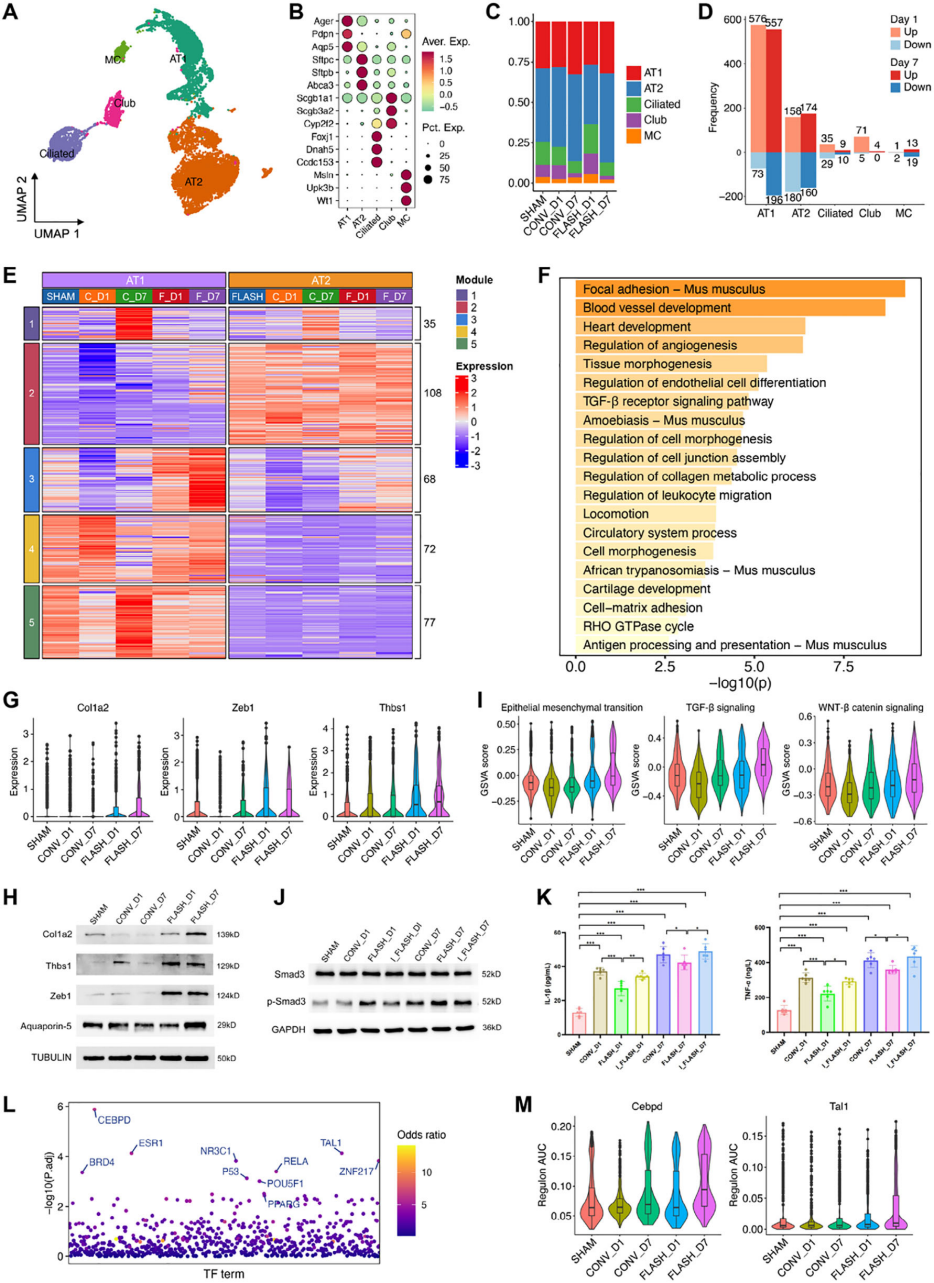
AT1 cells exhibited a strong repair response following FLASH irradiation. A) UMAP projection of 9745 epithelial cells identified, colored by cluster identities. B) Expression of selected marker genes in each epithelial cell subtype. C) Bar plots showing the fraction of cells from different clusters in each group. D) Bar plot denoting the number of DEGs in each cell type when comparing cells from the FLASH group to those from the CONV group. E) Heatmap of DEGs between FLASH and CONV groups in AT1 and AT2 cells. To minimize interference from minimally varying genes, only those with an average log2FC >0.5 or <−0.5 were included. F) The top 20 significantly signaling pathways of genes in the module 4, analyzed using Metascape. G) Violin plots showing expression levels of selected markers in AT1 cells from different groups. H) WB results showing the levels of Col1a2, Thbs1, Zeb1, and Aquaporin‐5 in the lung tissues of mice in each group after irradiation (*n* = 3). I) Violin plots showing GSVA scores of selected pathways in AT1 cells from different groups. J) WB results showing the levels of Smad3 and p‐Smad3 in the lung tissues of mice in each group after irradiation (*n* = 3). K) Bar graphs showing the levels of IL‐1β (left) and TNF‐α (right) in the lung tissues of mice in each group after irradiation (*n* = 6). ^*^ *p* <0.05, ^**^ *p* <0.01, ^***^ *p* <0.001. L) Dot plot depicting the enriched TFs regulating the expression of the module 4 signature. M) Violin plots showing regulon AUC values of selected TFs in AT1 cells from different groups. For each embedded boxplot in the violin plots, the box represents the interquartile range, the horizontal line in the box indicates the median, and the whiskers represent 1.5 times the interquartile range.

Pulmonary epithelial cells are crucial for tissue homeostasis and express various pattern recognition receptors that detect damage‐associated molecular patterns during RILI.^[^ ^53^ ^]^ We subsequently performed focused analyses of these cells, identifying 9745 cells in the dataset. Five distinct cell types were identified based on canonical lineage markers: alveolar type 1 (AT1) and type 2 (AT2) cells, ciliated cells, club cells, and mesothelial cells (**Figure** **7**A,B). No significant differences in epithelial cell composition were observed between groups, except for a slight decrease in the proportion of AT2 cells at 1 dpi post‐FLASH irradiation (Figure 7C). We then explored transcriptome changes in each cell type exposed to different irradiation treatments. DEGs were identified by comparing cells from the respective time‐point groups. This analysis revealed that FLASH irradiation induced substantial transcriptome changes in alveolar epithelial cells, particularly in AT1 cells, compared to CONV irradiation (Figure 7D; Figure S10A, Supporting Information).

To explore specific signatures and biological processes under different treatment conditions, we performed clustering analysis on DEGs in AT1 and AT2 cells, identifying five modules with distinct expression patterns (Figure 7E). Modules 1 and 5, which were highly expressed at 7 dpi post‐CONV irradiation in AT1 cells, were enriched for the terms “‘myeloid leukocyte migration”’ and “‘regulation of body fluid levels”’, respectively (Figure S10B, Supporting Information), indicating their activity in leukocyte chemotaxis and adaptive responses to irradiation injury. Module 2, predominantly expressed in AT2 cells, was enriched in “intracellular signaling cassette” and “cellular response to chemical stress” (Figure S10B, Supporting Information), highlighting the specific response of AT2 cells to stress. Module 3, composed of 68 genes, showed strong expression in the FLASH irradiation group, with increased expression from 1 to 7 dpi (Figure 7E). Functional enrichment analysis identified pathways related to inflammation suppression and tissue repair, including “focal adhesion”, “tissue morphogenesis”, and “TGF‐β receptor signaling pathway” (Figure 7F). Key TGF‐β and EMT markers in Module 3, including Col1a2, Zeb1, and Thbs1, were more highly expressed in the FLASH group compared to the SHAM and CONV groups, as confirmed by WB analysis (Figure 7G,H; Figure S11A, Supporting Information). Further assessment of hallmark pathways in single cells, including epithelial‐mesenchymal transition (EMT), TGF‐β signaling, and WNT‐β catenin signaling, confirmed higher activity in the FLASH group particularly at 7 dpi (Figure 7I). These pathways have been reported to play a critical role in shutting off inflammation and promoting tissue regeneration during the progression of pulmonary fibrosis.^[^ ^54^, ^55^ ^]^ Their activation in AT1 cells suggests an enhanced repair response following FLASH irradiation. To validate this, TGF‐β signaling inhibition was performed, and WB analysis showed a significant reduction in phosphorylated Smad3 (p‐Smad3) levels following TGF‐β inhibition (Figure 7J; Figure S11B, Supporting Information), confirming effective pathway suppression. Additionally, ELISA assays revealed a significant increase in pro‐inflammatory cytokines TNF‐α and IL‐1β in the FLASH group following TGF‐β inhibition (Figure 7K), confirming their role in regulating both inflammation and tissue repair after FLASH irradiation.

To investigate the underlying regulators of the AT1 cell repair process, we examined TFs enriched in module 3. This analysis identified CCAAT/enhancer‐binding protein delta (Cebpd) as the most significantly enriched TF (Figure 7L). Cebpd has been shown to modulate the EMT pathway^[^ ^56^ ^]^ and TGF‐β signaling^[^ ^57^ ^]^ and to promote cell migration and invasion by regulating ECM genes.^[^ ^58^ ^]^ Another enriched TF, T‐cell acute lymphoblastic leukemia 1 (Tal1, a.k.a. SCL), plays a prominent role in hematopoietic development and angiogenesis.^[^ ^59^ ^]^ Both Cebpd and Tal1 were enriched in the promoter regions of *Col1a2*, *Zeb1*, and *Thbs1* (Table S4, Supporting Information). The SCENIC analysis further revealed stronger activity of Cebpd and Tal1 regulons, suggesting their potential contribution to the repair process following FLASH irradiation through the regulation of TGF‐β signaling in AT1 cells (Figure 7M). Together, these findings demonstrate the repair response in AT1 cells following FLASH irradiation and underscore the crucial roles of key regulators in modulating this process.

## 3 Discussion

Although the FLASH effect was first observed in vivo as a sparing effect on lung tissue,^[^ ^15^ ^]^ how FLASH irradiation preserved this protective effect remains largely unknown. In this study, we systematically investigated the differential immune responses induced by CONV and FLASH irradiation in lung tissue using scRNA‐seq, covering >130 000 single cells collected from lung tissues representing acute injury stages of RILI. Our data provide a comprehensive characterization of cellular composition, immune activation, and tissue repair dynamics following both irradiation modalities. We found that FLASH irradiation significantly mitigated RILI compared to CONV, leading to reduced inflammation, enhanced immune activation, and accelerated tissue regeneration. These findings are novel in their identification of specific immune cell populations, particularly Ccrl2^+^ neutrophils and CD4^+^ CD40L^+^ Th cells, which play critical roles in mediating the distinct inflammatory and repair processes observed between FLASH and CONV irradiation. This study introduces a new paradigm for the application of FLASH therapy as a more effective treatment modality for minimizing radiation‐induced damage while promoting better lung tissue repair.

Immune cell populations exhibit the highest radiosensitivity to both FLASH and CONV irradiation, particularly lymphocytes, as observed in this study. Notably, B cells and NK cells, essential components of the immune system's innate defense, show a more pronounced reduction at 1 dpi under FLASH irradiation compared to CONV irradiation. However, these populations undergo substantial recovery by 7 dpi, suggesting that the observed depletion is transient rather than indicative of sustained immunosuppression. While FLASH irradiation has been reported to spare normal tissues across multiple organ systems, several studies have also reported FLASH‐induced lymphopenia in certain contexts.^[^ ^60^, ^61^ ^]^ This discrepancy could be attributed to tissue‐specific differences in lymphocyte turnover dynamics. For example, it has been reported that FLASH irradiation to the heart or spleen results in more pronounced lymphocyte depletion compared to CONV irradiation, with effects persisting for over three weeks.^[^ ^60^ ^]^ In contrast, studies on the hematopoietic system have shown that while both FLASH and CONV irradiation induce early lymphopenia, T lymphocytes in the FLASH group exhibit a higher degree of recovery by day 28.^[^ ^61^ ^]^ These findings, together with our data, suggest that FLASH‐induced lymphocyte depletion and recovery kinetics may vary across different tissues. Transcriptomic analysis reveals that FLASH irradiation also triggers a stronger early response at the transcriptional level, as evidenced by the greater number and higher fold changes of DEGs at 1 dpi. However, as the inflammatory response progresses, the number and extent of DEGs in the FLASH‐exposed group decrease significantly at 7 dpi, suggesting that FLASH‐induced transcriptional changes are transient and reversible. In contrast, CONV irradiation appears to create a more persistent inflammatory environment,^[^ ^62^, ^63^, ^64^ ^]^ with continued transcriptional alterations at later time points. The differential recovery kinetics observed between FLASH and CONV irradiation underscore a significant advantage of FLASH‐RT in minimizing long‐term immune activation and fostering faster immune reconstitution.

The biological implications of FLASH‐induced B and NK cell depletion remain complex. On one hand, B cells play a regulatory role by producing IL‐10, and their reduction could lead to prolonged inflammation and delayed tissue repair.^[^ ^65^ ^]^ Similarly, NK cells contribute to immune modulation and epithelial regeneration,^[^ ^66^ ^]^ raising the possibility that their depletion might influence lung injury resolution. On the other hand, excessive activation of B cells can amplify inflammation through TNF‐α and IL‐6 production,^[^ ^67^ ^]^ while NK cell‐mediated cytotoxicity may exacerbate tissue damage.^[^ ^68^ ^]^ Thus, FLASH‐induced reductions in these immune cell populations may, paradoxically, help prevent excessive inflammation and protect lung tissue. Importantly, our results demonstrate that by 7 dpi, B and NK cell populations in FLASH‐irradiated lungs have largely recovered, indicating that their initial depletion is transient rather than indicative of long‐term immunosuppression. The progression of RILI involves an extended inflammatory phase, followed by late‐stage pulmonary fibrosis. Given the dual roles of B and NK cells in inflammation and tissue repair, further studies are warranted to investigate whether FLASH affects their long‐term functional recovery and contributions to immune homeostasis post‐irradiation.

Neutrophils are among the first immune cells to respond to radiation‐induced damage, initiating an inflammatory cascade that can either resolve injury or exacerbate tissue damage.^[^ ^69^, ^70^ ^]^ In our study, neutrophils exhibited significantly increased infiltration at 7 dpi in both FLASH and CONV groups, with a notably greater increase in the CONV group, suggesting a more pronounced inflammatory response. Furthermore, CONV irradiation induced a significant increase in the Ccrl2^+^ neutrophil subset, a population associated with the promotion of pro‐inflammatory cytokines such as *Nfkbia*, *Ccrl2*, and *Sod2*, leading to a severe inflammatory milieu. In contrast, FLASH resulted in a more restrained neutrophilic response. Our trajectory and gene co‐expression analysis further suggest that the Ccrl2^+^ population represents terminally mature neutrophils. Key transcription factors, such as Irf5, Relb, and Gabpb1, were identified to regulate neutrophil maturation and activation. These transcription factors play critical roles in controlling the inflammatory response and have been implicated in the modulation of neutrophil polarization and function.^[^ ^34^, ^71^ ^]^ This insight into neutrophil dynamics suggests that targeting neutrophil infiltration or their inflammatory pathways could serve as a potential therapeutic strategy for mitigating radiation‐induced tissue damage, particularly in the context of CONV irradiation therapies, where inflammation plays a key role in chronic injury.^[^ ^72^ ^]^

T cells play a central role in the pathogenesis and resolution of RILI by secreting specific cytokines that modulate both the inflammatory response and tissue remodeling in the lung.^[^ ^73^, ^74^ ^]^ Our analysis revealed distinct transcriptional profiles in T cell subpopulations following FLASH and CONV irradiation. Specifically, FLASH irradiation induced transcriptomic changes associated with epithelial repair and tissue maintenance, whereas CONV irradiation was primarily linked to cytokine‐mediated signaling and immune cell recruitment. Among the various T cell subsets, CD4^+^ CD40L^+^ Th cells exhibited a particularly pronounced response. These cells are characterized by high expression of co‐stimulatory molecules such as *Cd40lg*, *Icos*, and *Cd28*. Both irradiation modalities led to an increasing trend in CD4^+^ CD40L^+^ Th cells, with a more significant expansion following FLASH irradiation. The interaction between CD40 and CD40L is crucial for modulating immune responses, particularly by activating APCs.^[^ ^75^, ^76^ ^]^ Furthermore, our analysis highlighted an additional role for CD4^+^ CD40L^+^ Th cells in regulating anti‐inflammatory responses through interactions with myeloid cells. This underscores the pivotal role of CD4^+^ CD40L^+^ Th cells in balancing inflammation and tissue repair following irradiation exposure. Further experimental validation is needed to confirm these observations and better understand the long‐term implications of CD4^+^ CD40L^+^ Th cell activity in FLASH‐RT.

In this study, we observed distinct differences in the recruitment of monocytes between CONV and FLASH irradiation. In the CONV group, we observed a significant increase in Mefv^+^ monocytes at 7 dpi, reflecting an intense inflammatory response, consistent with previous findings.^[^ ^77^ ^]^ In contrast, FLASH irradiation resulted in a more modest increase in monocyte recruitment, likely due to faster tissue recovery and a less sustained inflammatory milieu. CCC analysis revealed enhanced interactions between Mefv^+^ monocytes and other innate immune cells after CONV irradiation, particularly mediated by CCL and FN1 ligand‐receptor signaling. These interactions were less pronounced following FLASH irradiation, suggesting that FLASH not only limits monocyte recruitment but also dampens their immunomodulatory activity. Interestingly, although macrophage infiltration decreased in both groups, no significant difference was observed between the irradiation modalities regarding overall macrophage numbers within this timeframe, except for higher M1 polarization of AMs in the CONV group. This suggests that while initial monocyte recruitment is modality‐dependent, subsequent differentiation into macrophages might not predominantly occur within the first 7 dpi. Further investigation incorporating observation periods beyond 7 days is necessary to fully elucidate the long‐term effects of both irradiation modalities on macrophage dynamics.

The alveolar epithelium, a key component of alveoli, is pivotal for lung structure maintenance and function performance.^[^ ^78^ ^]^ Our study provides important insights into the response of epithelial cells, particularly AT1 cells, to radiation‐induced inflammation. AT1 cells are traditionally recognized for their role in the gas exchange of oxygen and carbon dioxide. Recent research has highlighted their involvement in coordinating alveolar immunologic activity.^[^ ^79^, ^80^ ^]^ Notably, in FLASH‐treated mice, we observed increased expression of genes related to EMT and TGF‐β signaling in AT1 cells. These pathways are well‐known for their dual role in tissue repair and fibrogenesis. On one hand, early activation of TGF‐β and EMT can promote inflammation resolution and tissue regeneration by enhancing cell migration, inhibiting cell death and senescence, and supporting cell dedifferentiation.^[^ ^54^, ^55^ ^]^ On the other hand, sustained or excessive TGF‐β and EMT activity has been implicated in fibroblast activation, extracellular matrix deposition, and ultimately pulmonary fibrosis.^[^ ^81^, ^82^, ^83^, ^84^ ^]^ In our study, the early activation of these pathways after FLASH irradiation coincided with reduced inflammation, suggesting an acute shift toward tissue repair and homeostasis. However, we acknowledge that our investigation was limited to the acute phase (up to 7 dpi), and it remains uncertain whether the observed TGF‐β and EMT activation is transient or may persist, thereby increasing the risk of long‐term fibrosis. Future studies with extended follow‐up and comprehensive evaluation of fibrosis are needed. Another limitation is that although we performed TGF‐β inhibition to validate its role, the inhibition was conducted at the tissue level rather than specifically in AT1 cells. While our findings suggest that TGF‐β signaling regulates repair responses in alveolar epithelial cells, future studies using cell‐type‐specific inhibition strategies (e.g., Cre‐lox systems or targeted delivery methods) are essential to determine the precise role of TGF‐β signaling in AT1 cells.

In our cell‐cell communication analysis, we did not detect significant direct interactions among Ccrl2^+^ neutrophils, CD4^+^ CD40L^+^ Th cells, Mefv^+^ monocytes, and epithelial cells, nor substantial differences in their interactions between FLASH and CONV irradiation. This suggests these immune responses might be independent rather than forming a direct cascade. Given that FLASH induces less overall tissue damage, each cell type may respond individually to the altered microenvironment rather than being directly regulated by other immune or epithelial cells.^[^ ^85^ ^]^ The reduction of neutrophils and monocytes in FLASH likely reflects a lower inflammatory burden rather than suppression by Th cells or epithelial cells.^[^ ^86^ ^]^ Similarly, the increase in CD4^+^ CD40L^+^ Th cells may represent an adaptive immune shift rather than direct regulation of neutrophils or epithelial repair.^[^ ^87^ ^]^ Moreover, it is important to acknowledge that the cell‐cell communication analysis detects primarily ligand‐receptor interactions, which may not fully capture cytokine‐mediated signaling, metabolic shifts, or ECM remodeling. To further explore these relationships, future studies involving functional depletion models and multiplex cytokine analysis will be necessary to determine potential indirect regulatory interactions.

Our study shows that FLASH irradiation induces a distinct immune response in normal lung tissue, featuring a transient inflammatory phase and more refined immune activation compared to CONV irradiation. These findings suggest that FLASH may modulate immune responses differently in antitumor radiotherapy. FLASH‐treated lungs exhibited reduced neutrophil and monocyte accumulation and diminished pro‐inflammatory signaling, key contributors to chronic tissue damage and fibrosis. This implies that FLASH not only protects normal lung tissue from excessive inflammation but also shapes immune dynamics in a way that could influence tumor progression. Neutrophils, known for both promoting and suppressing tumors, form extracellular traps (NETs) that support tumor growth and immune evasion.^[^ ^88^, ^89^ ^]^ The observed reduction in neutrophil‐related inflammation may foster a more favorable immune environment by limiting excessive neutrophil activation. Furthermore, FLASH irradiation increased CD4^+^ CD40L^+^ Th cells, critical for enhancing antigen‐presenting cell function,^[^ ^90^ ^]^ B cell activation,^[^ ^91^ ^]^ and CD8^+^ T cell priming^[^ ^92^ ^]^—key elements of an effective antitumor response. Thus, FLASH may not only mitigate normal tissue damage but also preserve or enhance adaptive immune functions contributing to tumor control. By balancing reduced neutrophil‐driven inflammation with enhanced CD4^+^ T cell activation, FLASH may offer an optimal balance between normal tissue protection and antitumor immunity. However, further studies are required to fully validate these observations and investigate their implications for tumor treatment.

Moreover, to establish a foundational understanding of FLASH‐induced immune modulation, this study focuses on normal lung tissue, thereby isolating the direct effects of FLASH without the confounding influence of tumor presence. However, it is important to consider the potential influence of pre‐existing tumor‐driven inflammation on lung tissue responses. Tumor‐adjacent lung tissue often exhibits an altered immune microenvironment, shaped by both tumor‐secreted factors and immune cell recruitment.^[^ ^93^, ^94^ ^]^ These changes may impact the immune response to irradiation and, consequently, the balance between tumor control and normal tissue protection. Although previous studies have demonstrated that FLASH irradiation can modulate immune responses within tumors,^[^ ^95^ ^]^ its effects on adjacent lung tissue and the broader immune microenvironment remain insufficiently understood. Future investigations employing tumor‐bearing models or analyzing tumor‐adjacent lung tissue will be essential to comprehensively evaluate the clinical implications of FLASH in balancing normal tissue protection with effective tumor control.

While our study provides valuable insights into the immunomodulatory effects of FLASH‐RT in murine models, the extrapolation of these findings to humans requires cautious interpretation. FLASH‐RT is still in its early clinical stages, and although preliminary clinical findings are encouraging, the breadth of human data remains limited. Notably, the FDA‐approved FAST‐01 trial showed that FLASH‐RT effectively relieved bone metastasis pain in 10 patients with equal or lower toxicity than conventional RT.^[^ ^96^ ^]^ Another case reported minimal skin toxicity in a CD30⁺ T‐cell lymphoma patient treated with 15 Gy FLASH‐RT in 90 ms, despite prior severe reactions to conventional RT.^[^ ^97^ ^]^ These cases suggest that the tissue‐sparing effect observed in mice may extend to humans. However, the specific immune responses identified in our study, such as Ccrl2^+^ granulocyte infiltration, CD4^+^ CD40L⁺ Th cell modulation, and AT1 cell activation, have not yet been directly demonstrated in human subjects receiving FLASH‐RT. Further clinical and mechanistic studies are essential to confirm whether the cellular and molecular dynamics observed in murine models can be faithfully recapitulated in humans.

In summary, our study provides new insights into the immune and cellular mechanisms underlying the differential effects of FLASH and CONV irradiation in RILI. We demonstrate that FLASH irradiation induces a more controlled immune response, characterized by modulated Ccrl2^+^ neutrophil infiltration, elevated CD4^+^ CD40L^+^ Th cell activity, and enhanced epithelial repair. These findings highlight the therapeutic potential of FLASH‐RT in minimizing the deleterious effects of irradiation while promoting tissue regeneration compared to conventional RT. These findings, combined with the extensive scRNA‐seq datasets generated, offer valuable insights into the dynamic cellular landscape of RILI. They further underscore the differences in immune responses between FLASH and CONV irradiation treatments and would open novel therapeutic avenues for the management of RILI.

## 4 Experimental Section

### Ethics Statement

The study was approved by the institutional review board of the Beijing Institute of Radiation Medicine (protocol ID: IACUC‐DWZX‐2024‐520). The work submitted in this article was solely completed by the teams of Changzhen Wang and Guofu Dong, and it is original. Excerpts from others’ work were clearly identified and acknowledged within the text and listed in the list of references.

### Mice and Groups

Eight‐week‐old male C57BL/6J mice (mean body weight ± standard deviation: 20 ± 0.9 g) were purchased from SPF (Beijing) Biotechnology Co Ltd (Beijing, China). Mice were housed in five per cage under standard conditions: room temperature 22 ± 2 °C, a 12‐h light‐dark cycle (lights on from 09:00 to 21:00), and relative humidity 50–60%. The animals were fed ad libitum with standard food (SPF (Beijing) Biotechnology Co., Ltd, Beijing, China) and water. After 7 days of acclimatization, mice were randomly divided into five groups (*n* = 12 each): control, FLASH_D1 irradiated, FLASH_D7 irradiated, CONV_D1 irradiated, and CONV_D7 irradiated groups.

### Irradiation and Dosimetry

CONV and FLASH whole‐thorax irradiation was performed at a dose of 17.8 Gy using a 6 MeV electron beam from the UHDR vertical test platform at the Department of Engineering Physics, Tsinghua University. An irradiation field of 2.5 cm (craniocaudal) × 6 cm (lateral) was used to cover the whole thorax. The irradiation parameters were listed in Table S5 (Supporting Information). Ashland's Gafchromic^TM^ EBT3 films (Ashland Inc., Covington, KY, USA) were used for dosimetry in both FLASH and CONV irradiation. Prior to irradiation, the films were calibrated using a 6 MV beam from a uRT‐linac 506c linac (United Imaging, Shanghai, China). The films were scanned 24 h after exposure with an Epson Perfection V850 Pro scanner. For each mouse, the entrance dose was recorded using EBT3 films placed directly on the thorax of mice during irradiation. The average entrance dose in the central 2.5 cm × 6 cm region was recorded as the delivered dose.

### Hematoxylin and Eosin (H&E) Staining and Masson's Trichrome Staining

Following euthanasia by cervical dislocation, lung tissues were collected from the mice. The left lung was fixed, dehydrated, and paraffin‐embedded. Then the sections were cut into 4‐µm‐thick sections for H&E staining and Masson's trichrome staining. The morphology of the lung tissue was observed under a 100× microscope. (OLYMPUS BX41; Olympus Corporation, Japan). The degree of pulmonary inflammation was assessed semi‐quantitatively using the Szapiel scoring system, defined as follows: score 0: no alveolitis; score 1: mild alveolitis with localized mononuclear cell infiltration, involving <20% of lung area, with largely intact alveolar structure; score 2: moderate alveolitis with 20–50% lung involvement; and score 3: severe alveolitis and pulmonary fibrosis, involving >50% of the lung, accompanied by mononuclear cells and/or hemorrhages in alveolar spaces, forming solid lesions. The cumulative score was used to represent the overall pathological injury level.

### Respiratory Function Tests

At 1 and 7 dpi, the pulmonary function of the mice was monitored using Buxco^®^ FinePointe^TM^ Whole Body Plethysmography 4‐Site System (Data Sciences International, DSI). Mice (*n* = 10) were placed smoothly in the chambers, and the data were analyzed after 15 min of recording using Buxco FinePointe software (DSI). The frequency (F), tidal volume (TV), minute volume (MV), inspiratory time (Ti), expiratory time (Te), mid‐expiratory flow (EF50), peak inspiratory flow (PIF), and peak expiratory flow (PEF) were determined.

### Immunofluorescence (IF) and Antibodies

Mouse lung tissues were embedded in an OCT compound and sectioned into 8‐µm‐thick slices using a cryostat (CM 1950, Leica, Germany). Sections were washed thrice with 1 × PBS (4 °C, 5 min per wash), then blocked with 10% bovine serum albumin (BSA) for 1 h at room temperature (RT) under light‐protected conditions. Subsequently, sections were incubated with primary antibodies diluted in 3% BSA overnight at 4 °C. After rewarming at RT for 1 h, sections underwent three additional PBS washes (4 °C, 5 min each). Fluorescent secondary antibodies were applied for 1 h at RT. Finally, sections were mounted with a DAPI‐containing medium and imaged using a fluorescence microscope (ECLIPSE CI‐L, Nikon). Images were acquired via NikonMosaic 1.6 software and analyzed using ImageJ. Primary and secondary antibodies for immunofluorescence are listed in Table S6 (Supporting Information).

### ELISA Assay

Tumor necrosis factor‐α (TNF‐α) and interleukin‐1β (IL‐1β) levels in mouse lung tissues were measured using ELISA kits (Shanghai Jianglai Biotechnology Co., Ltd., China), following the manufacturer's instructions. Absorbance was recorded with a SpectraMax iD3 microplate reader (Molecular Devices, USA).

### Western Blotting (WB)

Tissues were lysed in RIPA buffer, and proteins were isolated by centrifugation (12000 × g, 15 min, 4 °C). Protein concentrations were determined using a BCA assay kit (Thermo Fisher Scientific, USA). Equal protein quantities were diluted in RIPA buffer, denatured in 1 × sample buffer via boiling, and separated by SDS‐PAGE. Proteins were electrophoretically transferred to PVDF membranes, blocked with 5% non‐fat milk (1 h, room temperature), and incubated with primary antibodies overnight at 4 °C. After washing, membranes were incubated with HRP‐conjugated secondary antibodies (1 h, room temperature). Signals were visualized using an ECL 1228 Plus system (Lablead, China) and FluorChem R imager (Protein Simple, USA). Band intensities were quantified with ImageJ. Antibody details are provided in Table S7 (Supporting Information).

### Sorting of Mouse Type I Alveolar Epithelial Cells

A dissociation enzyme mixture was prepared using Collagenase II (V900892, Sigma–Aldrich Corporation, USA), DNase I (D5025, Sigma–Aldrich Corporation, USA), Dispase II (4942078001, Hoffmann‐La Roche AG, Switzerland), CaCl₂ (M1028, Sigma–Aldrich Trading Co.Ltd, China), 2% FBS (10099141C, GIBCO, USA), and RPMI 1640 (C11875500BT, GIBCO, USA). Washed lung tissue was minced into 1‐mm^3^ fragments in a culture dish containing 30 mL of the enzyme mixture. The mixture was transferred to a 50 mL tube and incubated at 37 °C for 60 min with agitation. Post‐digestion, the cell suspension was filtered through a 40 µm strainer, and the filtrate was collected. The strainer was rinsed with 10 mL RPMI 1640 (supplemented with 2% FBS), and the rinse solution was pooled with the filtrate. Cells were pelleted, resuspended in 4 mL of 1 × PBS containing 0.04% BSA, and prepared for downstream analysis.

Cells isolated from one lung were stained with biotinylated antibodies (5 µL each) in 500 µL complete DMEM containing 5 µL DNase. Antibodies included: anti‐CD45 (hematopoietic cells), anti‐CD16/32 (alveolar macrophages), anti‐CD31 (endothelial cells), anti‐Ter119 (erythroid cells), anti‐integrin β4 (club cells/distal lung progenitors), and anti‐SPC (type II alveolar epithelial cells) for lineage depletion. Cells were incubated on ice for 45–60 min with gentle flicking every 10 min. Dynabeads (65601, Thermo Fisher Scientific, USA) were pre‐washed twice with 1 mL D‐PBS (14190144, Thermo Fisher Scientific, USA) to remove azide. Post‐staining, cells were washed in 10 mL complete DMEM (11965‐092, Thermo Fisher Scientific, USA; 300 × g, 10 min, 4 °C), resuspended in 500 µL complete DMEM with 5 µL DNase, and mixed with pre‐washed beads in a 1.5 mL tube. Tubes were rotated in a cold room for 30 min to prevent bead sedimentation. For magnetic separation, tubes were placed in a magnetic stand for 2 min. Unbound cells were transferred to a new tube, and magnetic depletion was repeated twice (three rounds total). Lineage‐positive cells adhered to the tube wall, while unbound cells were retained. Antibody details are listed in Table S8 (Supporting Information).

### CD40L and TGF‐β Inhibition Assay

Eight‐week‐old male C57BL/6J mice (mean body weight ± standard deviation: 20 ± 0.9 g) were purchased from SPF (Beijing) Biotechnology Co., Ltd. (Beijing, China). After 7 days of acclimatization, mice were randomly divided into seven groups (*n* = 10 each) in each of the two inhibition experiments: SHAM, FLASH_D1 irradiated, FLASH_D7 irradiated, CONV_D1 irradiated, CONV_D7 irradiated, FLASH_D1 inhibitor‐irradiated, and FLASH_D7 inhibitor‐irradiated groups. Six hours prior to irradiation, mice in the FLASH_D1 and FLASH_D7 inhibitor‐irradiated groups (from two distinct inhibition experiments) were injected with CD40L (A2112, Selleck Chemicals LLC, USA) or SIS3 HCl (S7959, Selleck Chemicals LLC, USA). CD40L was used to block CD154–CD40 interactions on T helper cells, while SIS3 HCl inhibited Smad3 phosphorylation to suppress the TGF‐β signaling pathway. The respective inhibitors were re‐administered every other day post‐irradiation to sustain blockade efficacy.

### Single‐Cell Sequencing (scRNA‐Seq) Library Construction

After surgical collection, tissues were washed twice with 1× PBS, minced into 1mm^3^ fragments with sharp scissors, and enzymatically digested with collagenase II (2 mg mL^−1^, Sigma V900892) and DNase I (10 µg mL^−1^, Sigma DN25‐100MG) at 37 °C for 1 h. Then, the single cell suspensions were collected through a 40 µm strainer (MACS® SmartStrainer 40µm, Miltenyi Biotec, Germany), centrifuged at 500 g for 5 min, and resuspended with 1mL of 1 × PBS containing 0.04% weight/volume BSA after removal of the supernatant. Finally, the cell number and viability were assessed by an automatic cell counter. scRNA‐seq libraries were constructed using the 10x Genomics Chromium platform 3′ library and Gel Bead Kit V3.1 according to the manufacturer's instructions, with an estimated 10 000 single cells per sample. Next‐generation sequencing was performed on an Illumina Novaseq 6000 sequencer with a paired‐end 150 bp (PE150) strategy, with a minimum sequencing depth of 100 000 reads per cell.

### Computational Analysis of scRNA‐Seq Data

The Cell Ranger pipeline from 10x Genomics was employed to align reads to the mouse genome (mm10), quantify gene expression in single cells, and generate unique molecular identifier (UMI) count matrices for each sample. Further processing of the expression matrices was performed using the Seurat package (V4)^[^ ^98^ ^]^ in R. During quality control (QC), genes expressed in fewer than three cells were excluded, and cells were filtered based on the following criteria: i) ≤ 1000 or ≥ 60 000 total UMIs, ii) ≤ 500 or ≥ 8000 transcribed genes, or iii) ≥ 10% of UMIs mapped to mitochondrial genes. The expression matrices from different samples were then merged, log‐normalized, and scaled in Seurat's standard workflow. For principal component analysis, the union of the top 1000 variable genes from each sample was selected, excluding ribosomal, immunoglobulin, and mitochondrial genes to minimize unwanted variation. Batch effects were corrected using the Harmony algorithm.^[^ ^99^ ^]^ Cell clusters were identified via the shared nearest neighbor modularity optimization algorithm, and marker genes for each cluster were determined using the Wilcoxon rank‐sum test. Clusters were visualized using Uniform Manifold Approximation and Projection (UMAP) for dimensionality reduction.

Additionally, Scrublet^[^ ^100^ ^]^ was used to detect potential doublets in the dataset. Doublet scores for single cells were calculated per sample using default settings, with thresholds adjusted after visual inspection of the score distributions. Clusters with a doublet rate exceeding 10% were designated as doublet clusters and excluded from further analysis.

### Developmental Trajectory Inference

The Monocle (V3) algorithm was utilized to infer cell differentiation trajectories, following the methodology described previously.^[^ ^30^ ^]^ The raw count matrix was used to construct Monocle 3′s CellDataSet object, excluding genes with <200 UMIs or expressed in <1% of cells within each cell type. Cell alignment was performed based on sample identity to minimize potential batch effects. Default parameters were applied for the subsequent dimensionality reduction.

### Diffusion Component Analysis

Diffusion component analysis was performed using the R package destiny (version 3.16.0)^[^ ^101^ ^]^ to infer the major non‐linear components of variation across cells. The normalized expression profiles of the top 100 markers for each cell cluster were used to construct the diffusion maps, and the top two diffusion components were selected for visualization.

### Cell–Cell Communication (CCC) Analysis

CellChat (Version 1.6.1) was used to infer the CCC process among different cell types as described.^[^ ^102^ ^]^ The CellChat object was constructed based on the normalized expression profile. Cellular communication probability was computed using the default parameters, with interactions involving <10 cells in each cell group filtered out. The communication probability of signaling pathways was estimated by aggregating the communication probabilities of all ligand‐receptor interactions associated with the corresponding pathways.

### Weighted Gene Co‐Expression Network Analysis (WGCNA)

The weighted gene co‐expression network was constructed with the R package hdWGCNA (Version 0.3.03).^[^ ^32^ ^]^ Genes expressed in ≥10% of cells were selected for network construction. The optimal soft threshold for adjacency was determined using a signed network to ensure a scale‐free topology. Harmonized module eigengenes (hMEs) were calculated to summarize the gene expression profiles within each co‐expression module. Pairwise correlations between genes and hMEs were used to estimate the eigengene‐based connectivity, facilitating the identification and ranking of hub genes.

### Functional Enrichment Analysis

Gene Ontology (GO) term enrichment analysis was performed in the R package clusterProfiler (version 4.10.0)^[^ ^103^ ^]^ to identify overrepresented biological processes among the differentially expressed genes. Functional annotations were conducted using Metascape,^[^ ^104^ ^]^ providing additional insights into pathways, molecular functions, and cellular components associated with the gene sets. All analyses were carried out using default parameters.

### SCENIC Analysis

The pySCENIC (version 0.12.1)^[^ ^33^ ^]^ analysis was conducted for neutrophils and epithelial cells. Co‐expression modules were derived from the normalized expression matrix exported from Seurat. Two transcription factor (TF) ranking databases, TSS ± 10 kb, and 500bpUp100Dw, were used to identify direct targets, which were subsequently pruned based on cis‐regulatory cues. A regulon‐by‐cell matrix was calculated to quantify the enrichment of each regulon as the area under the recovery curve (AUC) of genes defining the regulon. To detect differentially enriched regulons, we applied a linear model to the AUC matrix using the *lmFit* function from the limma package (version 3.58.1).^[^ ^105^ ^]^

### Transcription Factors (TFs) Identification

The Enrichr web tool^[^ ^106^ ^]^ was utilized to identify TFs that potentially regulate genes in epithelial cell gene modules. TF enrichment analysis was based on target gene sets curated in the ChEA Transcription Factor Targets Dataset (“ChEA 2022”).

### Statistical Analysis

All data from the validation experiments were expressed as mean ± standard error of the mean (SEM) and all graphs were generated using GraphPad Prism 8.0 software (San Diego, CA, USA). Data normality was tested using the Shapiro‐Wilk test. For normally distributed data, statistical significance was assessed using a two‐tailed Student's t‐test or one‐way analysis of variance (ANOVA), followed by Tukey's test for multiple comparisons. For non‐normally distributed data, non‐parametric tests were used. A *p* <0.05 was considered statistically significant (^*^ *p* <0.05, ^**^ *p* <0.01, ^***^ *p* <0.001).

## Conflict of Interest

The authors declare no conflict of interest.

## Supporting information



Supporting Information

Supplemental Tables

## Data Availability

The scRNA‐seq dataset generated in this study are available at the NCBI Gene Expression Omnibus database under the following accession number: GSE298566.
