# Supplementary material for: FLASH Irradiation Modulates Immune Responses and Accelerates Lung Recovery: A Single‐Cell Perspective
Source: Adv Sci (Weinh). 2025 Jun 19;12(34):e01797. doi: 10.1002/advs.202501797 (PMC12442706; doi:10.1002/advs.202501797)
Supplement: Supplementary file 1 — Supporting Information [file ADVS-12-e01797-s002.docx]

**Supplementary Material**

**FLASH Irradiation Modulates Immune Responses and Accelerates Lung Recovery: A Single-Cell Perspective**

*Hao Lu, Menghua Li, Cheng Quan, Caihui Li, Dawei Li, Zhihui Li, Jing Xu, Lihui Zhang, Qixiang Liu, Guofu Dong***, Changzhen Wang**

H. Lu, M. Li, C. Quan, C. Li, D. Li, Z. Li, J. Xu, L. Zhang, Q. Liu, G. Dong, C. Wang

Beijing Institute of Radiation Medicine

Beijing 100850, P.R. China

E-mail: dongguofu052@126.com (G. Dong); wangcz2002@aliyun.com (C. Wang).

H. Lu, M. Li, G. Dong, C. Wang

Anhui Medical University

Hefei 230032, China

H. Lu, M. Li, C. Quan, and C. Li contributed equally to this work.

**Supplementary Figures**


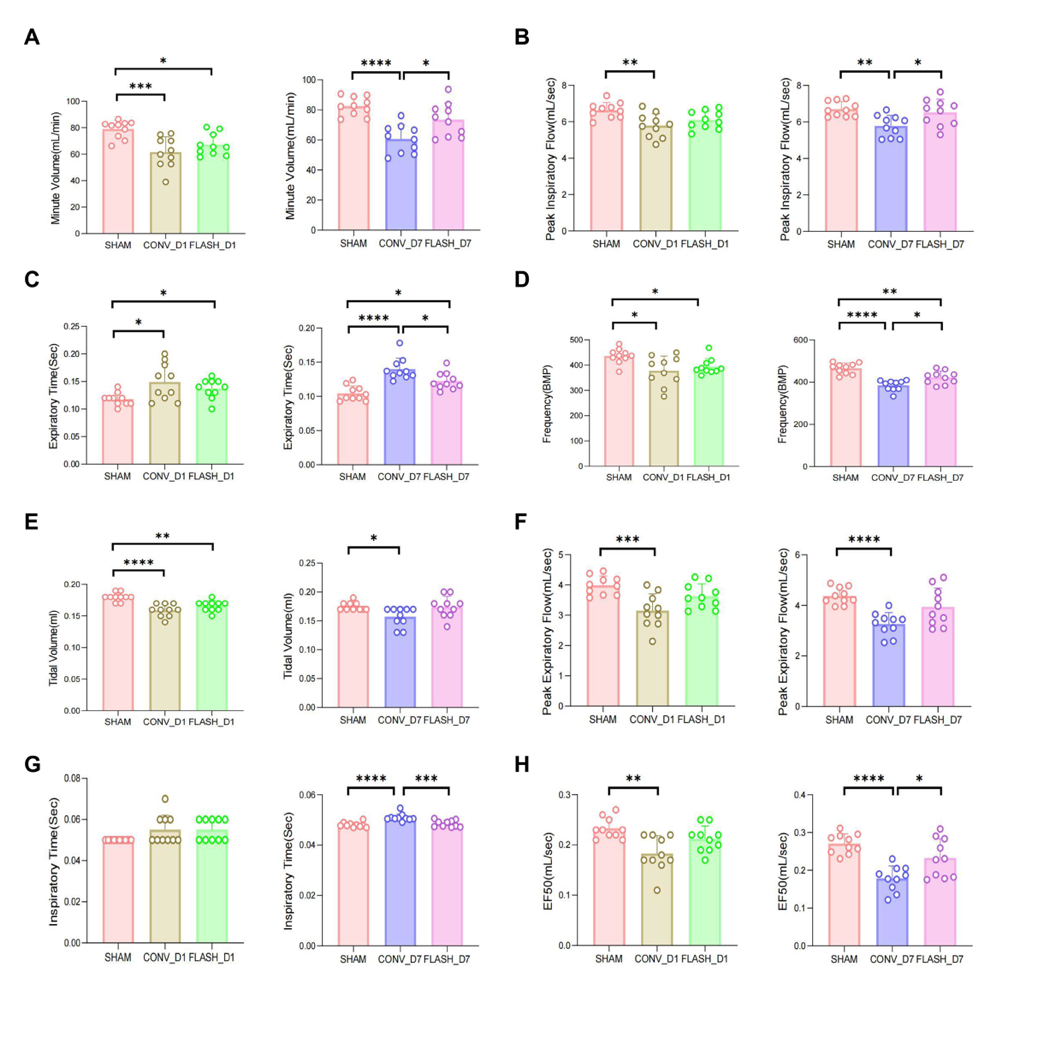


**Figure S1.** Comparison of pulmonary function between CONV and FLASH irradiated mice measured by whole-body plethysmography. A) Minute volume (MV), B) peak inspiratory flow (PIF), C) expiratory time (Te), D) frequency (F), E) tidal volume (TV), F) peak expiratory flow (PEF), G) inspiratory time (Ti), and H) mid-expiratory flow (EF50). **p* < 0.05, ***p* < 0.01, ****p* < 0.001, *****p* < 0.0001.

**
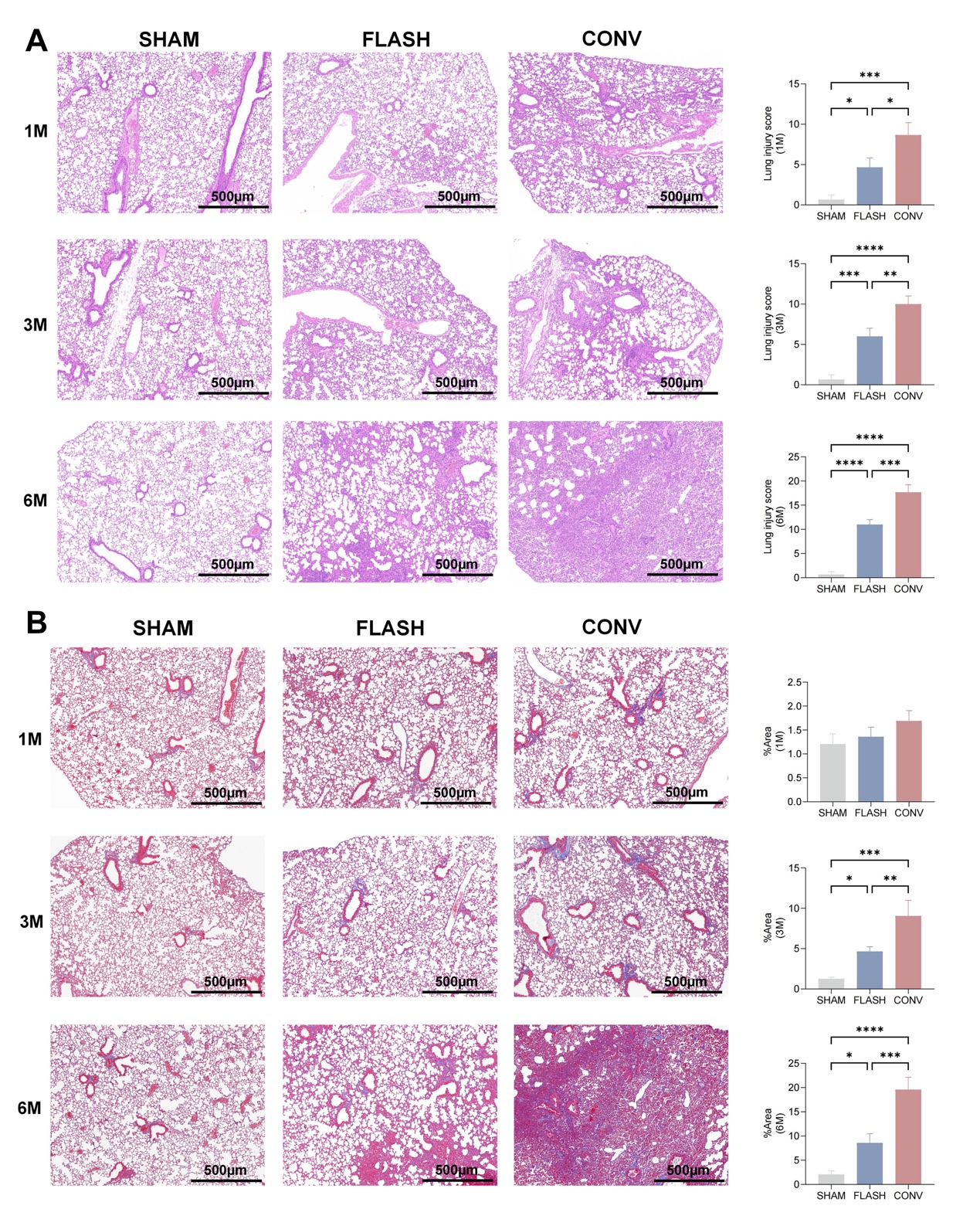
**

**Figure S2.** Histopathological staining results of mouse lung at different time points after 18 Gy irradiation. A) H&E staining of mouse lung tissue. The embedded bar plots show the results of the pathologic damage scores of lung tissues at 1, 3, and 6 months after irradiation (n = 3, scale bar = 500 μm). **p* < 0.05, ***p* < 0.01, ****p* < 0.001, *****p* < 0.0001. B) Masson staining of mouse lung tissue. The embedded bar plots show the results of semi-quantitative analysis of Masson staining of lung tissues at 1, 3, and 6 months after irradiation (n = 3, scale bar = 500 μm). **p* < 0.05, ***p* < 0.01, ****p* < 0.001, *****p* < 0.0001.


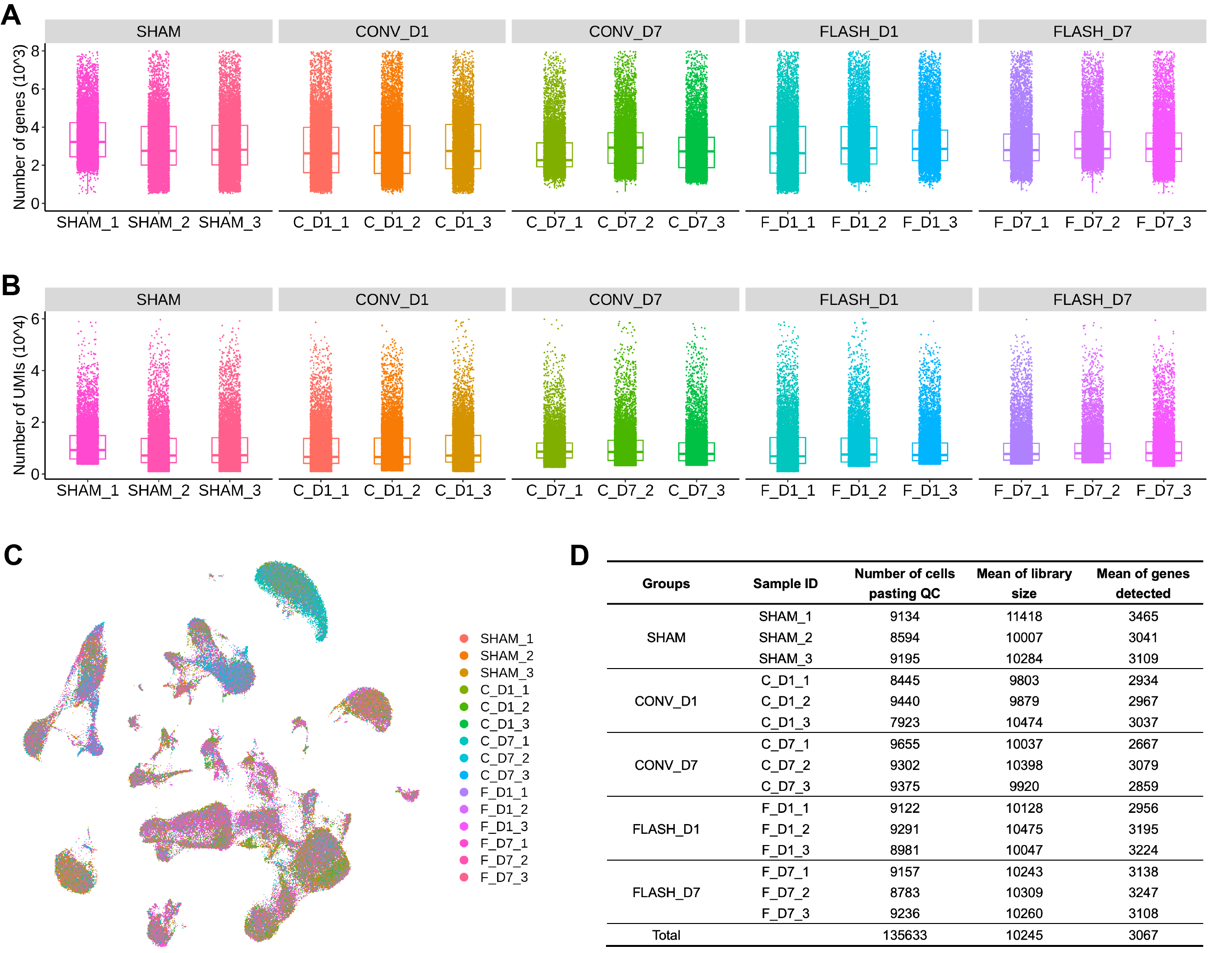


**Figure S3.** Quality control of scRNA-seq data for the lung cellular ecosystem post-CONV and FLASH irradiation. A,B) Boxplots showing the distribution of numbers of detected genes (A) and UMIs (B) in each sample. The box represents the interquartile range, the horizontal line in the box indicates the median, and the whiskers represent 1.5 times the interquartile range. C) UMAP projection of the 135,633 cells profiled, colored by samples. D) Quality control statistics of each sample for scRNA-seq data.


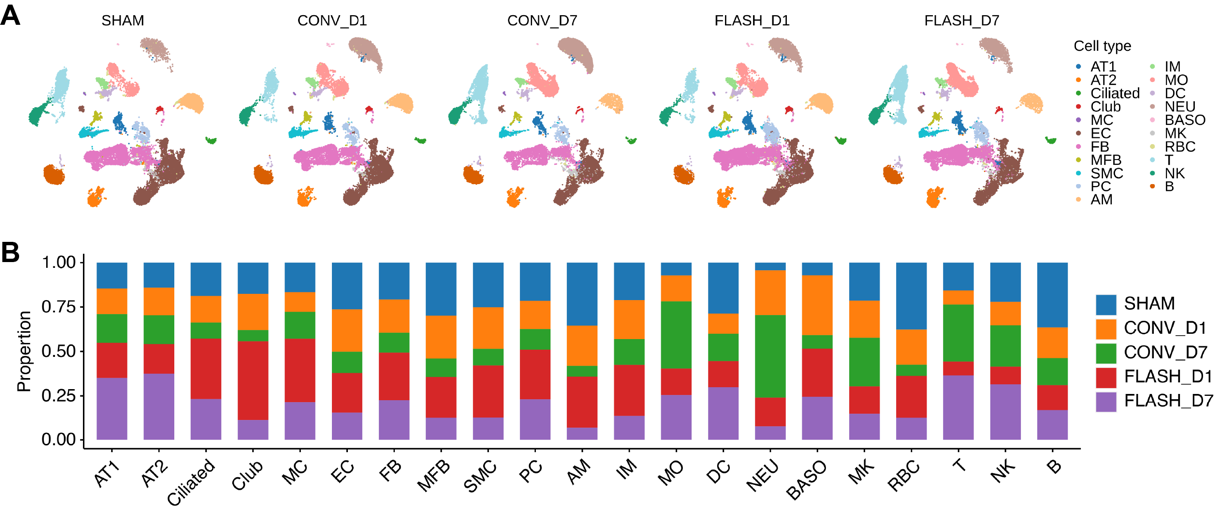


**Figure S4.** Dynamics of cell proportion in each group. A) UMAP projection of the 135,633 cells profiled, split by groups. B) Bar plots showing the fraction of cells originating from different groups in each cell type.


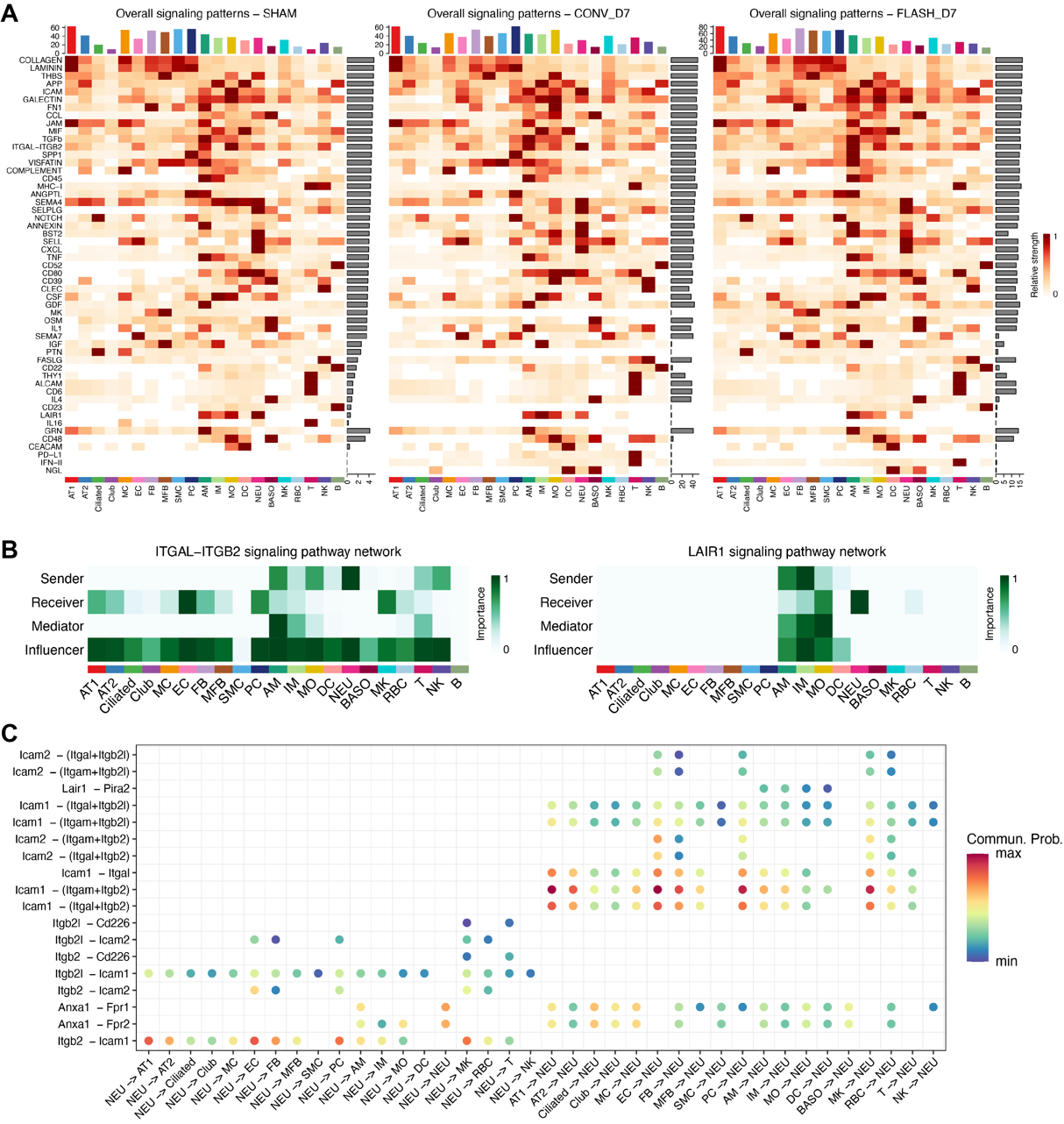


**Figure S5.** Comparison of signaling pathways associated with each cell population among SHAM, CONV, and FLASH groups. A) Heatmap showing the relative strength of overall signaling patterns associated with each cell population in SHAM (left), CONV (middle), and FLASH (right) groups at 7 dpi. Pathways in which neutrophils were not involved in any of the three groups are omitted. B) Heatmap depicting the relative importance of each cell type as sender, receiver, mediator, and influencer in ITGAL-ITGB2 and LAIR1 signaling pathways. C) Dot plot showing the significant ligand-receptor pairs associated with ICAM, ITGAL-ITGB2, ANNEXIN, and LAIR1 signaling pathways.


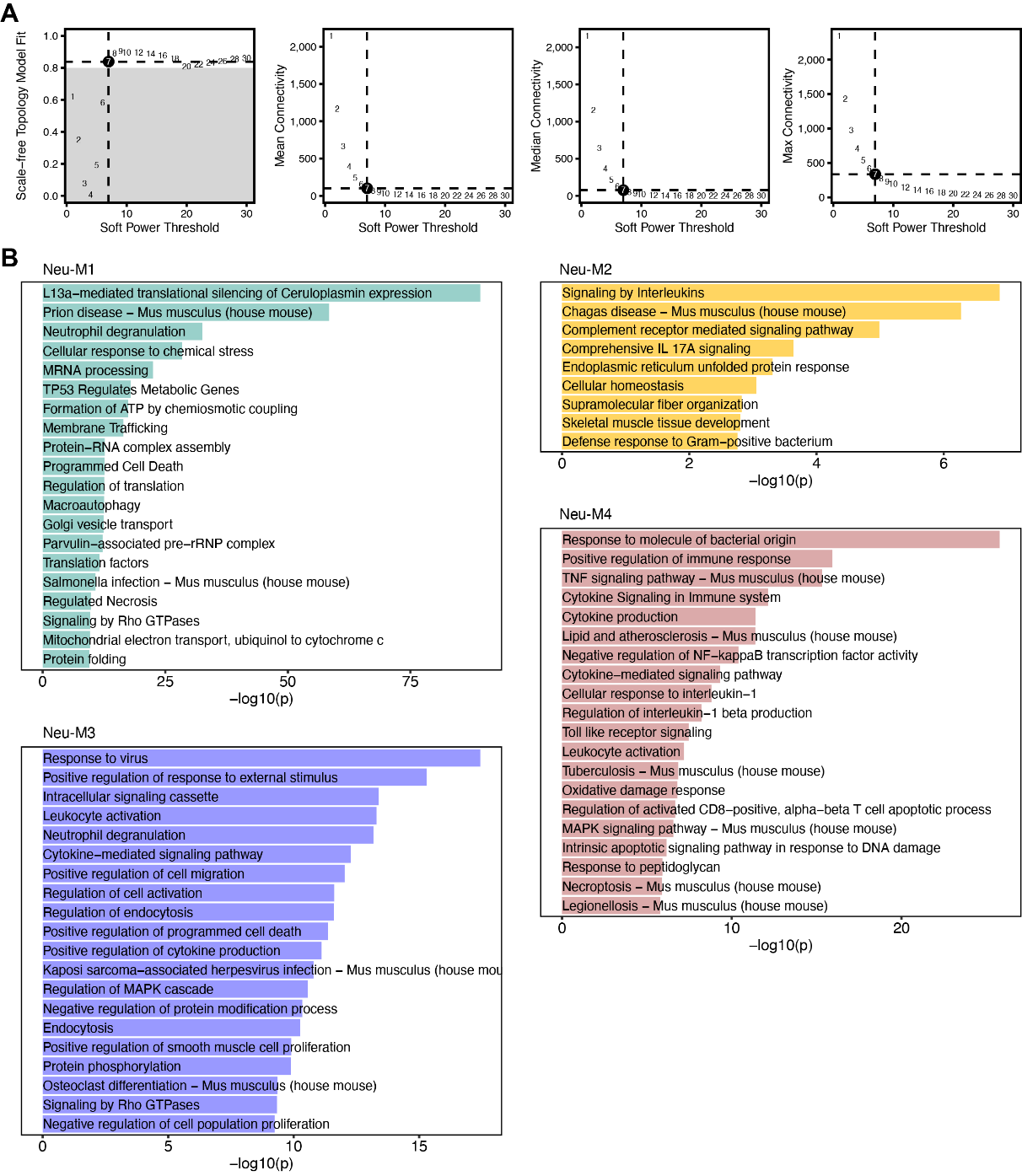


**Figure S6.** hdWGCNA identified gene modules in neutrophils. A) Analysis of the scale-free index, mean connectivity, median connectivity, and max connectivity (left to right) for various soft-threshold powers. B) The top 20 (or fewer) significantly signaling pathways of genes in each module, analyzed using Metascape.


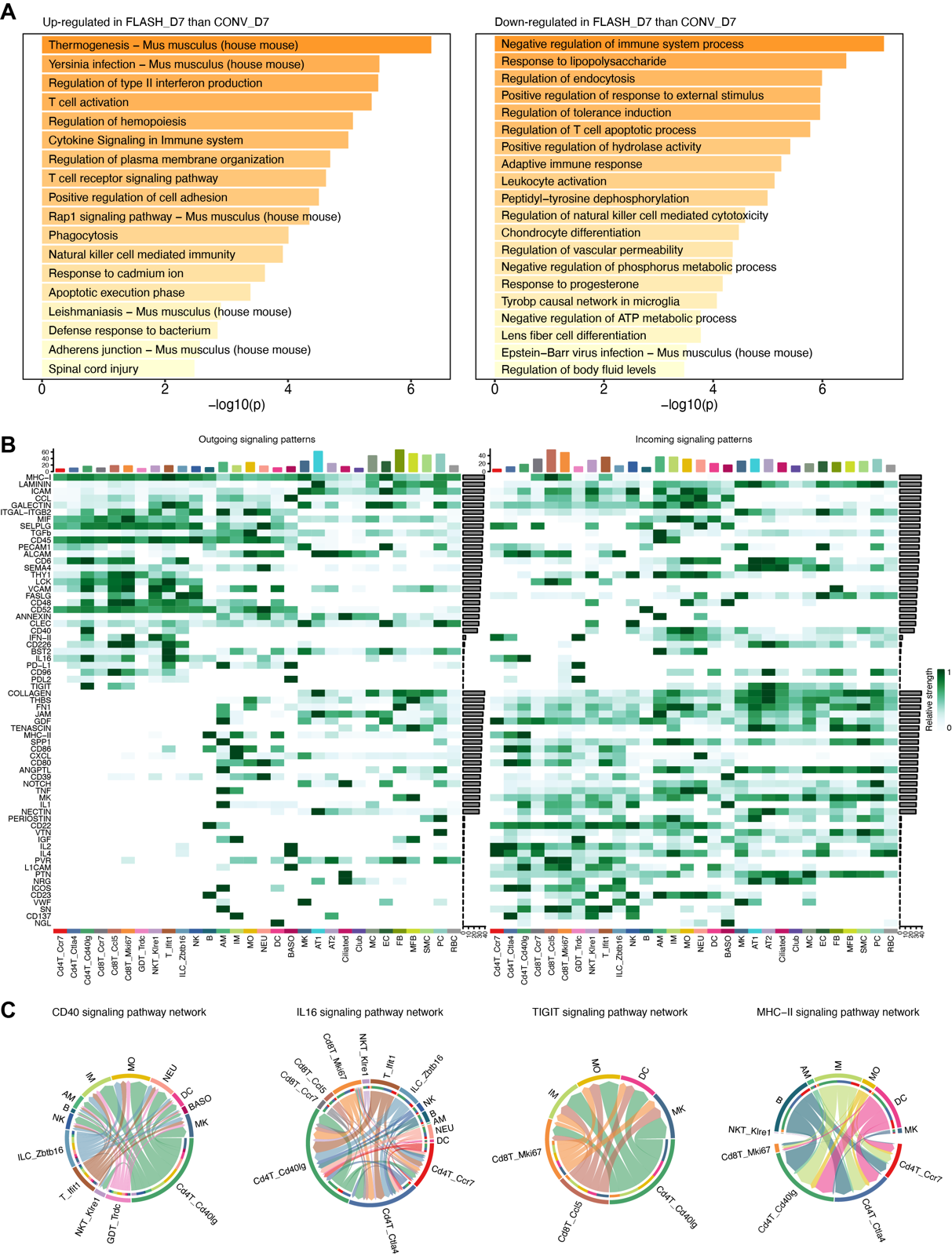


**Figure S7.** Functional annotation and cell-cell communication (CCC) analysis of T cells within the microenvironment of RILI. A) The top 20 significantly signaling pathways of genes up (left) or down (right) regulated in Ccl5^+^ T cells from the FLASH group compared to those from the CONV group at 7 dpi. Enrichment analyses were performed with Metascape. B) Heatmap showing the relative strength of outgoing (left) and incoming (right) signaling patterns associated with each cell population in the FLASH group at 7 dpi. C) Chord diagrams depicting the inferred signaling network of the selected signaling pathways in the FLASH group at 7 dpi.

**
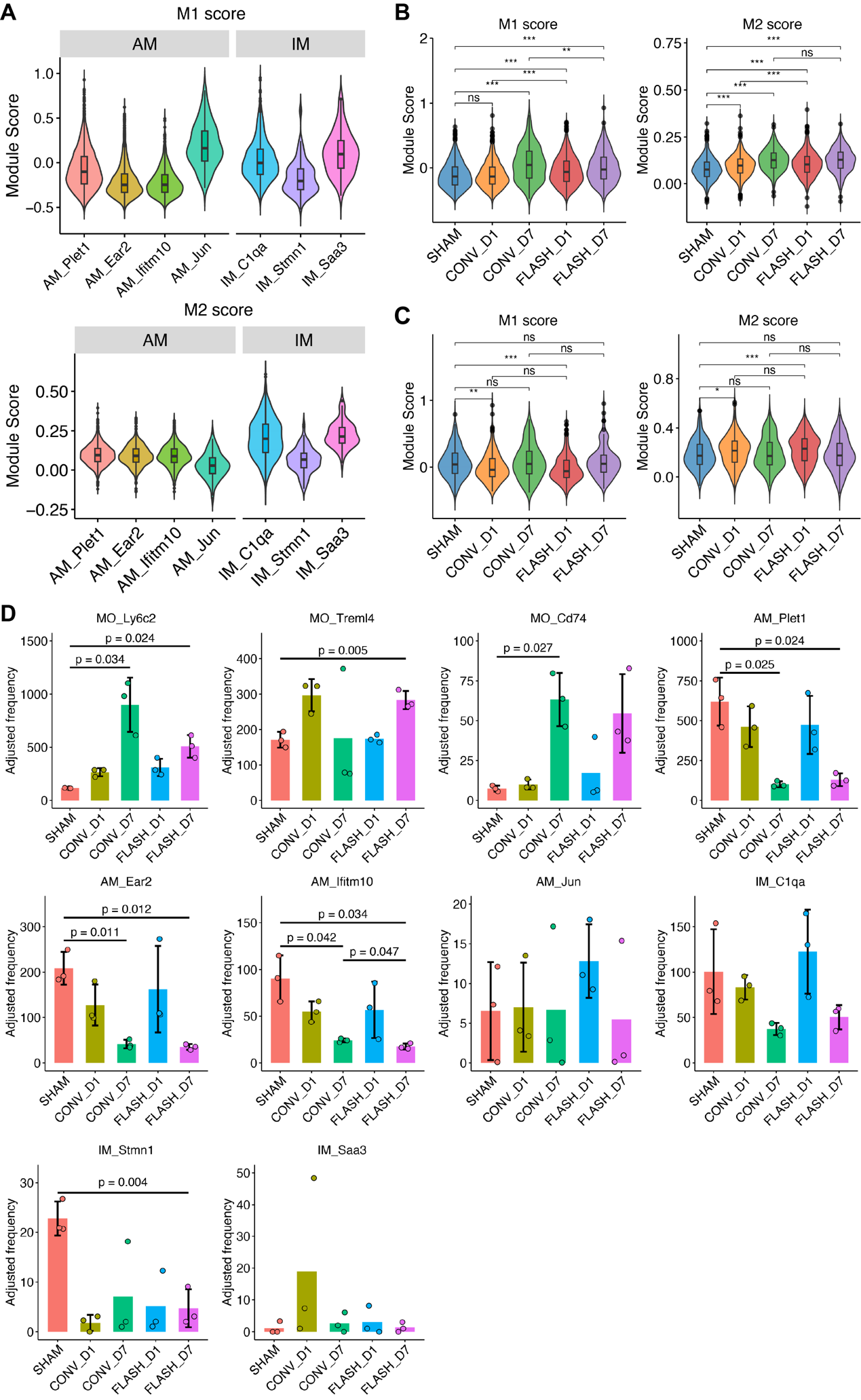
**

**Figure S8.** Estimation of polarization scores and comparison of cell numbers among different groups. A) Violin plots showing M1 and M2 polarization scores in AM and IM clusters. Module scores were estimated by AddModuleScore in Seurat. For each embedded boxplot, the box represents the interquartile range, the horizontal line in the box indicates the median, and the whiskers represent 1.5 times the interquartile range. B) Comparison of M1 (left) and M2 (right) polarization scores of AM_Plet1 among groups. ***p* < 0.01, ****p* < 0.001. C) Comparison of M1 (left) and M2 (right) polarization scores of IM_C1qa among groups. **p* < 0.05, ***p* < 0.01, ****p* < 0.001. D) The adjusted frequency of cells from each group within monocyte and macrophage clusters. Cell numbers were scaled to a total of 10,000 per sample.


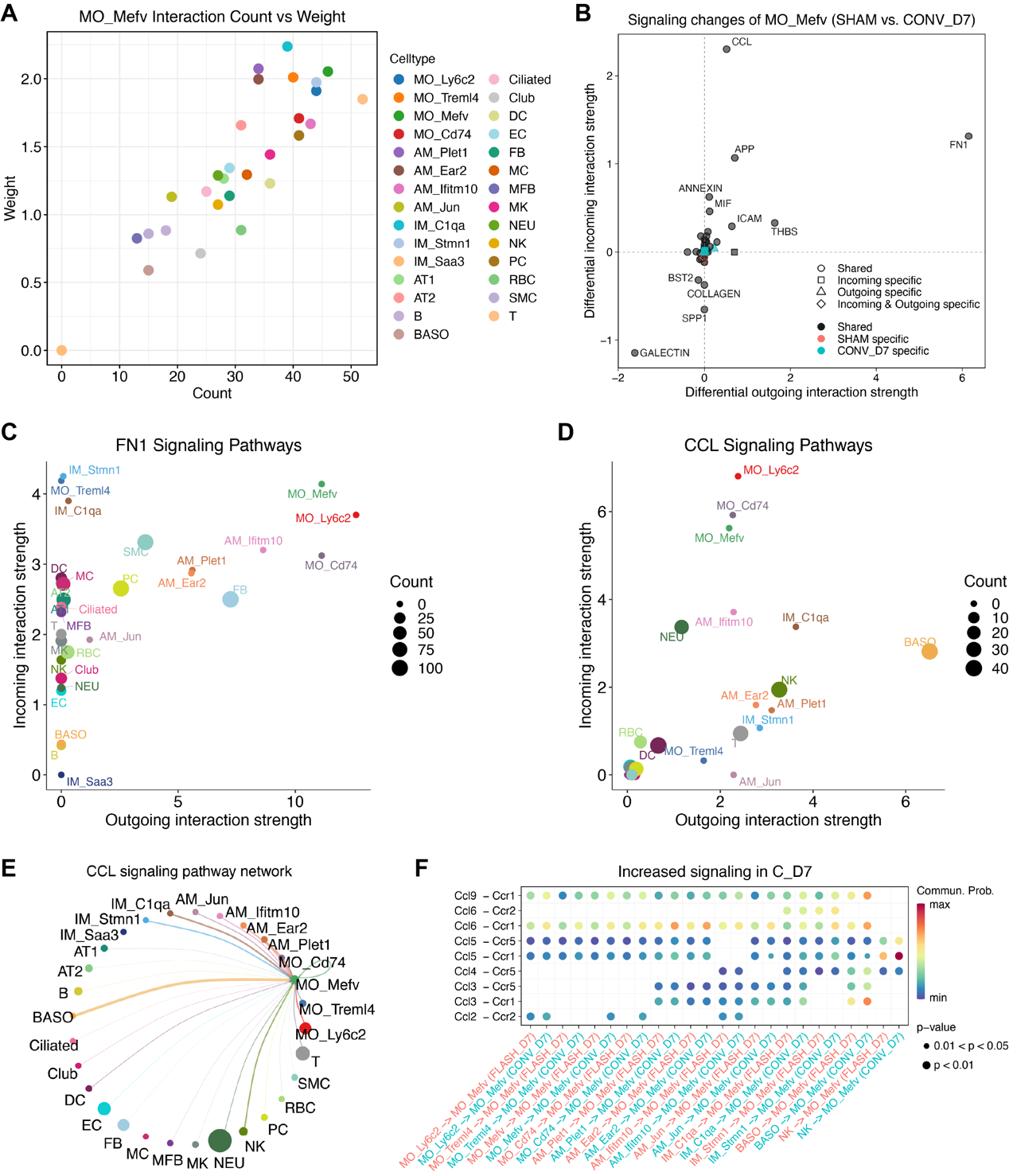


**Figure S9.** Cell-cell communication (CCC) analysis of monocyte- macrophages within the microenvironment of RILI. A) Dot plot showing the interaction counts and weights of MO_Mefv cells with other cell types. B) Dot plot showing the difference of CCC signaling pathways between the CONV_D7 and SHAM groups. C) Interaction counts of various cell types within the FN1 signaling pathways. D) Interaction counts of various cell types within the CCL signaling pathways. E) Dot plot showing the significant ligand-receptor pairs associated with CCL signaling pathways.


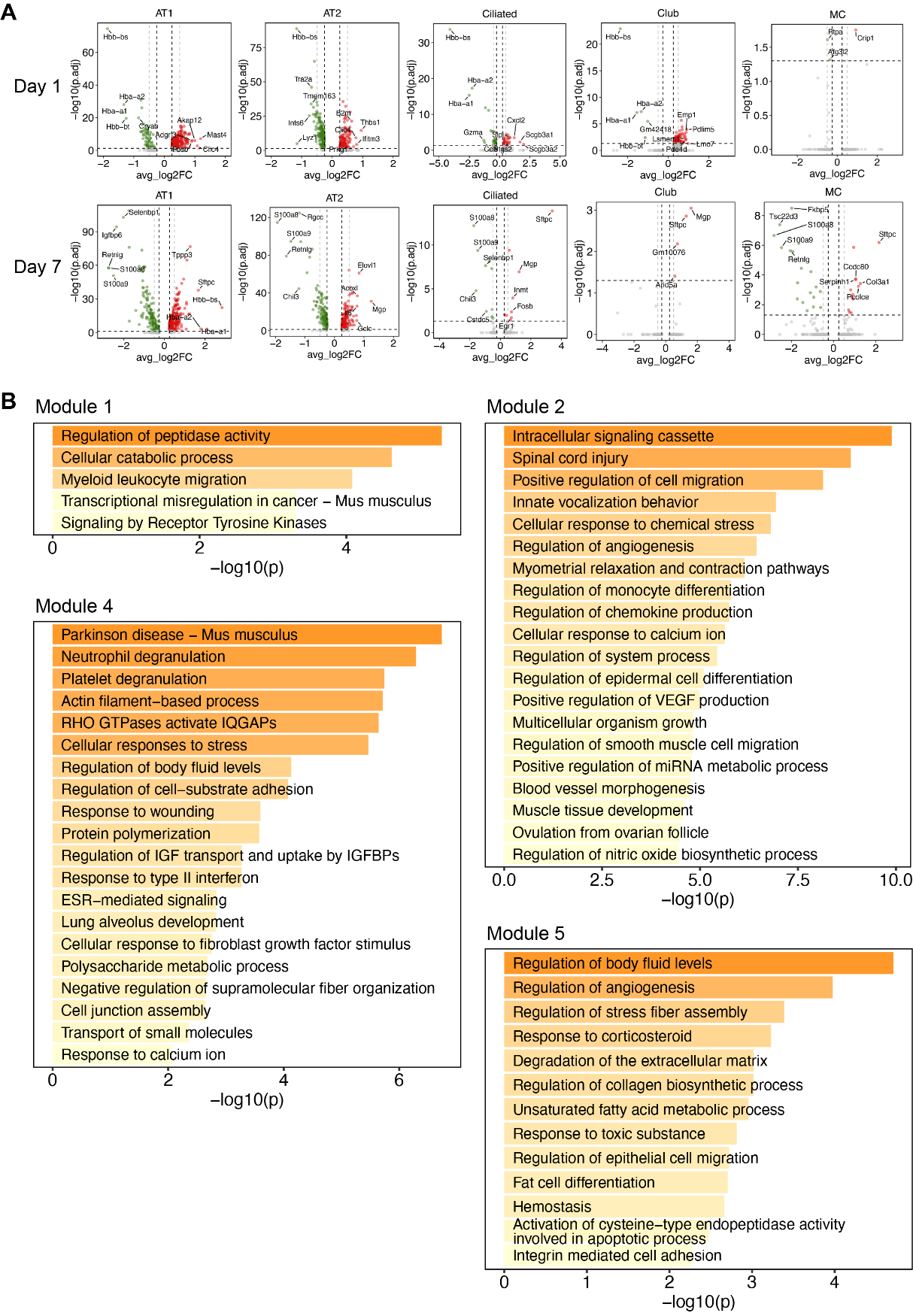


**Figure S10.** Differential expression analysis between FLASH and CONV irradiation in epithelial cell types. A) Volcano plots showing the differentially expressed genes (DEGs) between FLASH and CONV irradiation in each epithelial cell type. Vertical lines represent average log2FC thresholds at ±0.25 (black) and ±0.5 (gray), while horizontal lines indicate an adjusted p-value (p.adj) threshold of 0.05. B) The top 20 (or fewer) significantly signaling pathways of genes in the DEGs modules for AT1 and AT2 cells, related to the Figure 6B in the main text. Enrichment analysis was performed with Metascape.

**
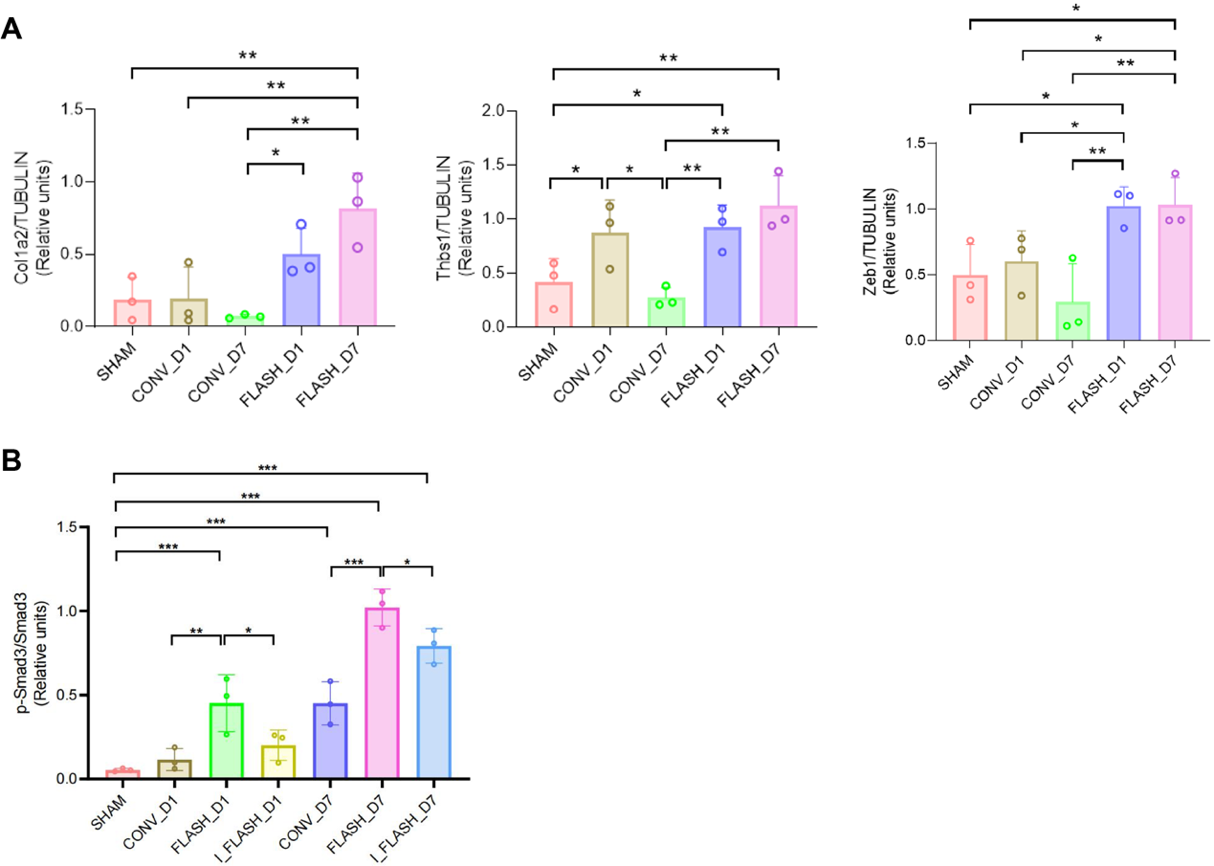
**

**Figure S11.** WB quantification of protein content in lung tissue of mice in each group after irradiation. A) Bar plots showing the relative expression levels of Col1a2, Thbs1, and Zeb1 proteins (left, center, and right, respectively) (n = 3). **p* < 0.05, ***p* < 0.01. B) Bar plots showing the relative expression levels of p-Smad3 protein (n = 3). **p* < 0.05, ***p* < 0.01, ****p* < 0.001.
